# Evidence for a rapid rate of molecular evolution at the hypervariable and immunogenic *Mycobacterium tuberculosis PPE38 *gene region

**DOI:** 10.1186/1471-2148-9-237

**Published:** 2009-09-21

**Authors:** Christopher RE McEvoy, Paul D van Helden, Robin M Warren, Nicolaas C Gey van Pittius

**Affiliations:** 1 DST/NRF Centre of Excellence for Biomedical Tuberculosis Research/MRC Centre for Molecular and Cellular Biology, Division of Molecular Biology and Human Genetics, Faculty of Health Sciences, Stellenbosch University, PO Box 19063, Tygerberg, South Africa

## Abstract

**Background:**

*PPE38 *(Rv*2352c*) is a member of the large *PPE *gene family of *Mycobacterium tuberculosis *and related mycobacteria. The function of PPE proteins is unknown but evidence suggests that many are cell-surface associated and recognised by the host immune system. Previous studies targeting other *PPE *gene members suggest that some display high levels of polymorphism and it is thought that this might represent a means of providing antigenic variation. We have analysed the genetic variability of the *PPE38 *genomic region on a cohort of *M. tuberculosis *clinical isolates representing all of the major phylogenetic lineages, along with the ancestral *M. tuberculosis *complex (MTBC) member *M. canettii*, and supplemented this with analysis of publicly available whole genome sequences representing additional *M. tuberculosis *clinical isolates, other MTBC members and non tuberculous mycobacteria (NTM). Where possible we have extended this analysis to include the adjacent *plcABC *and *PPE39/40 *genomic regions.

**Results:**

We show that the ancestral MTBC *PPE38 *region comprises 2 homologous *PPE *genes (*PPE38 *and *PPE71*), separated by 2 *esat-6 *(*esx*)-like genes and that this structure derives from an *esx/esx/PPE *duplication in the common ancestor of *M. tuberculosis *and *M. marinum*. We also demonstrate that this region of the genome is hypervariable due to frequent IS*6110 *integration, IS*6110*-associated recombination, and homologous recombination and gene conversion events between *PPE38 *and *PPE71*. These mutations result in combinations of gene deletion, gene truncation and gene disruption in the majority of clinical isolates. These mutations were generally found to be IS*6110 *strain lineage-specific, although examples of additional within-lineage and even within-cluster mutations were observed. Furthermore, we provide evidence that the published *M. tuberculosis *H37Rv whole genome sequence is inaccurate regarding this region.

**Conclusion:**

Our results show that this antigen-encoding region of the *M. tuberculosis *genome is hypervariable. The observation that numerous different mutations have become fixed within specific lineages demonstrates that this genomic region is undergoing rapid molecular evolution and that further lineage-specific evolutionary expansion and diversification has occurred subsequent to the lineage-defining mutational events. We predict that functional loss of these genes could aid immune evasion. Finally, we also show that the *PPE38 *region of the published *M. tuberculosis *H37Rv whole genome sequence is not representative of the ATCC H37Rv reference strain.

## Background

The *Mycobacterium tuberculosis *genome contains two large gene families that together comprise around 10% of its protein coding capacity [1]. These families, termed *PE *and *PPE*, appear to have originated in the fast growing mycobacterial species before undergoing extensive expansion and diversification in certain slow growing species, particularly *M. ulcerans*, *M. marinum *and members of the *M. tuberculosis *complex (MTBC) [2]. The large multi-protein families encoded by these genes are of unknown function, although reports suggest that at least some members are cell surface associated [3-6] and can be antigenic [4,5,7-9], a finding that has stimulated interest in their potential role in vaccine production, e.g. [10,11]. PPE proteins contain a proline-proline-glutamic acid (PPE) amino acid sequence at positions 7-9 in a highly conserved N-terminal domain of approximately 180 amino acids. The C-terminal domains of both PE and PPE protein families are highly variable in both size and sequence and often contain repetitive DNA sequences that differ in copy number between genes [1]. Several studies have shown that some *PE *and *PPE *genes are polymorphic and this has been interpreted as indicating strong selection pressure for antigenic variants that may aid in host immune evasion [3,7,12-17].

**Figure 1:**
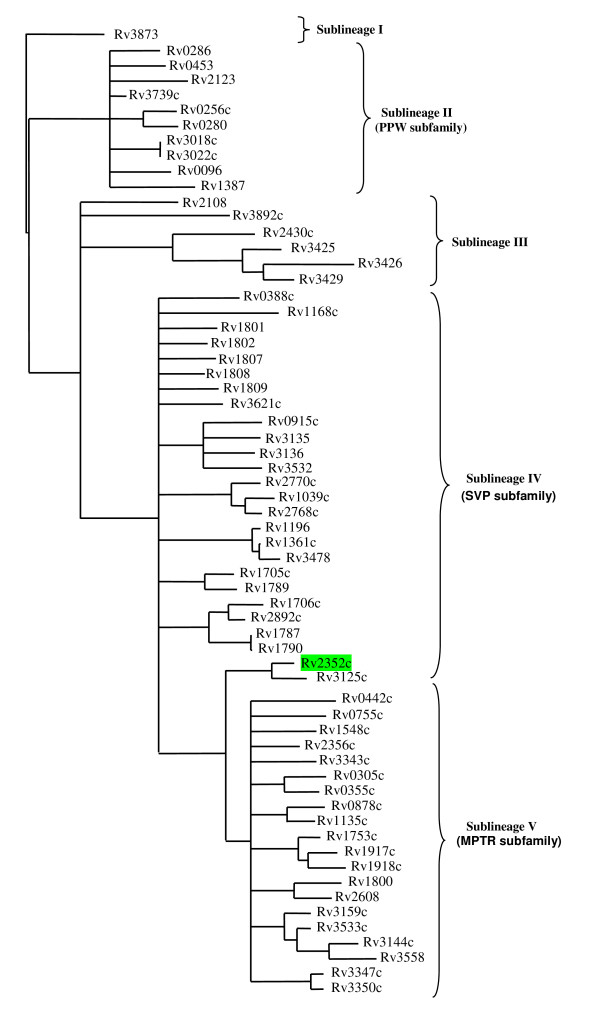
**Phylogenetic reconstruction of the evolutionary relationships between members of the H37Rv *M. tuberculosis *PPE protein family members**. The phylogenetic tree was constructed from a phylogenetic analysis done on the 180 aa N-terminal domains of the PPE proteins. Results show the division of PPE proteins into 5 sublineages with PPE38 (Rv2352*c*, highlighted in green) located at the border of sublineages IV (SVP subfamily) and V (MPTR subfamily). Reproduced from ref [2] with permission from the authors.

A recent phylogenetic analysis of the 69 *PPE *genes present in the *M. tuberculosis *reference strain H37Rv has uncovered their evolutionary relationships and reveals that they can be divided into several subfamilies [2] (Figure 1). *PPE38 *(Rv*2352c*) is shown to be a member of *PPE *sublineage IV (the SVP subfamily) and analysis of its protein sequence confirms that it encodes the SVP subfamily-defining amino acid sequence (GxxSVPxxW) at positions 309 - 317. However, along with the closely related gene *PPE49 *(Rv*3125c*), it shares a more recent common ancestor with PPE sublineage V members (the MPTR subfamily) than with any other member of the SVP sublineage (Figure 1). Although no reports are available regarding its antigenicity or other biochemical features, because of its position on the "border" of sublineages IV and V, *PPE38 *was included in a larger study aimed at determining the genetic variation of *PE *and *PPE *genes between various strains of *M. tuberculosis *(manuscript in preparation). Here we present our analysis of this gene and its surrounding region using a cohort of phylogenetically diverse and well-defined *M. tuberculosis *clinical isolates representing all of the major phylogenetic lineages, along with the most ancestral MTBC divergent member, *M. canettii*. This has been supplemented by *in silico *analysis of this genomic region in the whole genome sequences of 15 publicly available *M. tuberculosis *strains, 8 other MTBC members (*M. bovis*, *M. bovis *BCG, *M. microti*, 3 × *M. africanum*, dassie bacillus and oryx bacillus), and 14 non tuberculous mycobacteria (NTM) species (7 fast growing species and 7 slow growing species). We show that this region is hypervariable in MTBC members and that this has resulted in a rapid rate of genetic divergence occurring between most *M. tuberculosis *strain lineages.

## Results

### Identification of the variable *PPE38 *region and the RvD7 deletion

**Figure 2:**
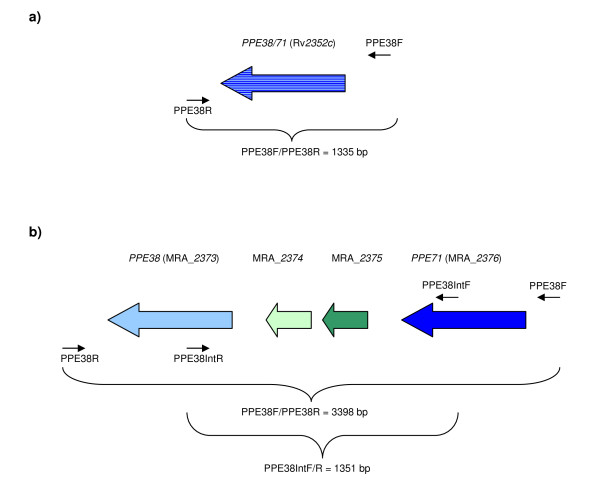
**Schematic representations of the *PPE38 *gene region in the H37 reference strain published sequences**. The *PPE38 *region from the published H37Rv (2a) and H37Ra (2b) sequences are shown. Colour coding as follows: *PPE38 *pale blue, *PPE71 *dark blue, MRA_*2374 *pale green, MRA_*2375 *dark green. Locations of the PPE38F/R and PPE38 IntF/R primers are shown. **2a. H37Rv ATCC reference strain (published whole genome sequence) **The published H37Rv sequence [1] represents the RvD7 genotype. Recombination between *PPE38 *and *PPE71 *results in a single *PPE38/71 *gene (Rv*2352c*) and loss of the 2 *esx*-like genes MRA_*2374 *and MRA_*2375*. The PPE38F/R primers (black arrows) are predicted to produce an amplicon of 1335 bp from the RvD7 genotype. It is impossible to determine which *PPE38/71 *gene has been deleted hence the mixture of colours used. The published H37Rv sequence is not representative of the H37Rv ATCC reference strain, most clinical isolates, or the H37Ra whole genome sequence [19]. This genotype is also seen in strains SAWC 2240 (CAS, F20), SAWC 1748 (Pre-Haarlem, F24), SAWC 1595 (Quebec/S), SAWC 1841 (Haarlem, F4), CPHL_A (WA-1, *M. africanum*), T17 (PGG1, EAI), EAS054 (PGG1, EAI), strain C (LCC, "3 bander") and Haarlem (PGG2, F4) [see additional file 1]. **2b. H37Rv ATCC reference strain (actual) and H37Ra (published whole genome sequence) **This represents the ancestral MTBC genotype that is also seen in *M. canettii*. It contains the 2 identical *PPE38 *(MRA_*2373) *and *PPE71 *(MRA_*2376*) genes separated by the 2 *esx*-like genes MRA_*2374 *and MRA_*2375*. Gene annotations are as reported for the H37Ra published sequence [19]. Locations of primers used for PCR and sequence analysis are indicated (black arrows). This is also the true genotype of the ATTC reference strain H37Rv.

The published *M. tuberculosis *H37Rv genome sequence [1] predicts the amplification of a 1335 bp PCR product spanning the entire *PPE38 *gene when using the PPE38F/R primer pair (Figure 2a, Table 1). However, our analysis of 3 *M. canettii *clinical isolates, the H37Rv and H37Ra American Type Culture Collection (ATCC) reference strains (ATCC numbers 25618 and 25177 respectively), and 40 *M. tuberculosis *clinical isolates from different IS*6110 *RFLP-defined strain lineages covering all three principal genetic groups (PGGs) [18], revealed that only 7 strains produced this amplicon. Most samples (including the H37Rv and H37Ra ATCC reference strains and the 3 *M. canettii's*) produced a dominant amplicon of approximately 3.4 kb, while other samples produced amplicons of alternate sizes varying from approximately 2.5 to 5 kb, and 3 samples failed to amplify. Analysis of the H37Ra whole genome sequence revealed that the 3.4 kb amplicon (actual predicted size = 3398 bp) results from the presence of a second copy of *PPE38 *along with 2 *esat-6 *(*esx*)-like genes (annotated as MRA_*2374 *and MRA_*2375 *in H37Ra) in this region (Figure 2b) [19]. The second copy of *PPE38 *has been previously identified and designated as *PPE71 *in the CDC1551 whole genome sequence [20]. Its coding region is identical to *PPE38 *and both genes also share the same 5'-untranslated region up to position -35 bp. As previously suggested by Zheng and colleagues [19], this genomic structure suggests that the published H37Rv sequence represents the result of a homologous recombination event between *PPE38 *and *PPE71 *that has deleted one of these genes along with MRA_*2374 *and MRA_*2375*. This deletion is annotated as RvD6 in their analysis of the H37Ra whole genome sequence [19]. However, the authors did not acknowledge that the term RvD6 was appropriated in 2005 to define a specific variation between the *M. bovis *and H37Rv genomes [21]. For purposes of clarity and uniformity we therefore propose that the this deletion rather be termed RvD7.

**Table 1:** Sequences of primers used for PCR amplification and sequencing.

**Primer name**	**Sequence (5' -- 3')**	**Comment**
PPE38F	TTTTCGGTGTGGATTGTCT	3398 bp amplicon for H37Ra-like genotype, 1331 bp amplicon for RvD7 genotype.
PPE38R	GCCAGGGATTTCCAACGAC	
PPE38IntF	ATGTCGGCGGAGTTGGGTAAG	1351 bp amplicon for H37Ra-like genotype, no product for RvD7 genotype.
PPE38IntR	TAGCCTGACCAGCCGACAACT	
21delF	GGGGATGATGCCGATGC	111 bp amplicon for wild-type genotype, 90 bp amplicon for 21del genotype.
21delR	ACACTGGGCCGAGCCTG	
IS5'	GGTACCTCCTCGATGAACCAC	IS*6110*-binding sequencing primer used to determine region upstream of IS*6110*.
Xho1	TTCAACCATCGCCGCCTCTAC	IS*6110*-binding sequencing primer used to determine region downstream of IS*6110*.
plcA5'	CAAATGTCCGGGACAAGG	Primes from the 5' region of *plcA*. Used to PCR and sequence the region between *plcA *and *PPE38 *in conjunction with the PPE38IntF primer in *M. canettii *isolates.

### Detailed analysis of the *PPE38 *region

In order to analyse the variation in this region more thoroughly, we designed additional primers (PPE38IntF/IntR, Table 1, Figure 2b) to allow PCR analysis and sequencing of the region between *PPE38 *and *PPE71*. Sequence analysis of the complete 3.4 kb product produced using the PPE38F/R primers in the H37Rv ATCC reference strain, one *M. canettii *and 4 of our clinical *M. tuberculosis *isolates (SAWC 974, SAWC 2666, SAWC 1870 and SAWC 300) confirmed its complete homology to the published sequence of H37Ra [19] apart from 3 SNPs observed in *M. canettii *and one SNP in isolate SAWC 1870 that are described below. The discrepancy between our H37Rv ATCC reference isolate and the published H37Rv sequence was further investigated by PCR analysis from DNA derived from three additional independent cultures of H37Rv from different sources, including one that had been newly purchased from the ATCC. In each case the H37Ra-like genotype (Figure 2b) was confirmed and not the published H37Rv RvD7 genotype (Figure 2a, data not shown). The two additional *M. canettii *isolates were also analysed with these PCRs and the H37Ra-like genotype was also confirmed (data not shown). A complete list of *PPE38 *genotypes representing all analysed samples, comprising H37Rv, H37Ra, all 40 *M. tuberculosis *clinical isolates from our cohort, 3 *M. canettii *clinical isolates plus 15 *M. tuberculosis *and 8 non-*M. tuberculosis *members of the MTBC (analysed *in silico *from publicly available whole genome sequences - see below), along with group, lineage (F) and mutation details is listed in additional file 1.

### Analysis of clinical isolates displaying alternate *PPE38 *region genetic structures and determination of lineage specificity

The *PPE38 *region of clinical isolates that produced PCR amplicons of sizes that did not correspond to the H37Ra-like genotype were analysed in more detail by sequencing PCR amplicons. In order to characterize IS*6110*-associated mutations, the IS5' and Xho1 primers were used (Table 1). Twelve isolates possessed IS*6110*-mediated mutations, with two of these also displaying indels involving presumably recombination-mediated swapping of parts of the 5' untranslated regions of *PPE38 *and *PPE71*. One isolate revealed a 5'-untranslated region indel without an accompanying IS*6110 *mutation. Four isolates displayed the RvD7 genotype as defined by the H37Rv whole genome sequence (Figure 2a). The final isolate failed to produce PCR product when using any of the *PPE38*-associated primer pairs, although PCRs directed at other regions of the genome were successful. We conclude that this isolate possesses a large deletion in the *PPE38 *region. Details and figures of the characterized mutations can be found in additional files 1 and 2 (S1 - S18). Additional clinical isolates were investigated in many cases in order to determine whether specific characterized mutations were IS*6110 *lineage-, cluster-, or isolate-specific. Results showed that in most cases the mutation was specific to all of the different clusters analysed from within the lineage, although several instances of within-lineage and even within-cluster variation was observed. Details of this analysis can be found in additional file 2 (S1 - S18).

### *In silico *analysis of the PPE38 region in *M. tuberculosis *and other MTBC member whole genome sequences

**Figure 3:**
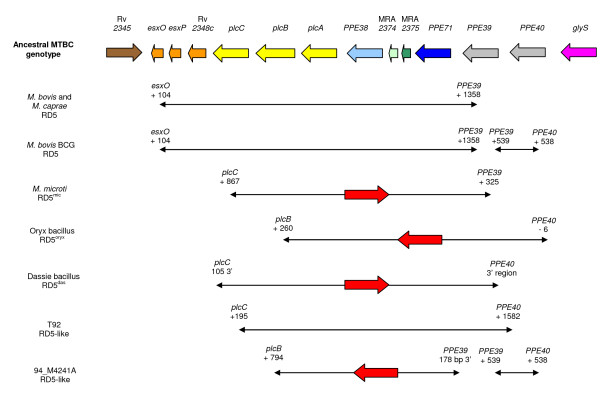
**RD5 and RD5-like deletions seen in MTBC isolates**. This region is susceptible to frequent large deletions. Here we show the genes surrounding *PPE38 *along with the deleted regions characterised in 5 non-*M. tuberculosis *MTBC members [22-25,32], along with the deletions detected in the *M. tuberculosis *whole genome sequences T92 and 94_M4241A. A red arrow indicates the presence and direction of IS*6110 *at a deletion point. Deletions caused by homologous recombination between *PPE39 *and *PPE40 *in *M. bovis *BCG and 94_M4241A are also shown. Numbering refers to gene nucleotide positions.

The results obtained from our clinical isolates encouraged us to further investigate the genomic structure of this region in isolates whose whole genome sequences are publicly available. Along with the H37Rv and H37Ra sequences previously described we also analysed the region in 13 *M. tuberculosis *and 6 non-*M. tuberculosis *MTBC members for which the whole genome sequences are publicly available. For convenience, although the dassie and oryx bacillus genomes have not been completed, we have included known information on their *PPE38 *regions [22,23] in this section, thus providing a total of 21 additional MTBC genomes for analysis. Surprisingly, only 4 of these genomes (H37Ra, CDC1551 and *M. africanum *isolates GM041182 and K85) displayed the "normal" (ancestral) H37Ra-like *PPE38 *genotype of 2 *PPE *genes separated by 2 *esx*-like genes (Figure 2b). Six genomes (including H37Rv) displayed the RvD7 genotype (Figure 2a). Six genomes displayed various IS*6110*-associated mutations that, in some cases, were associated with additional indel mutations. The remaining 7 genomes, including all of the non-human animal-associated organisms, displayed large RD5 and RD5-like [24,25] deletions that spanned the entire *PPE38 *region including adjacent genes. Details and figures of all the characterized mutations can be found in the additional files 1 and 2 (S19 - S32). A schematic representation of the 7 large RD5 and RD5-like deletions can be seen in Figure 3.

### Analysis of micro-mutations within the *PPE38/71 *gene sequences

Along with the macro-mutational events described above, the *PPE38 *region of 15 isolates from our cohort plus the fully sequenced genomes were also examined for mutations at the micro-mutational level. Apart from the 21del mutation which is described below, only 4 isolates (*M. canettii*, SAWC 1870, KZN 4207 and K85) were confirmed to possess micro-mutations. These are detailed in additional file 1. These results demonstrate that micromutations within the *PPE38 *region are rare.

### Analysis of the 21del mutation

**Figure 4:**
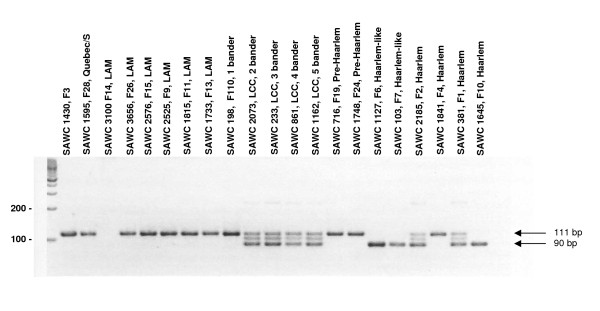
**21del PCR results for all 21 members of PGG2**. Wild type gene amplicon = 111 bp. 21del amplicon = 90 bp. Sample SAWC 3100 (F14) is negative for all *PPE38*-related PCRs suggesting complete deletion of this region [see additional file 2, S8]. In isolates that possess both a normal and a 21del gene copy an additional amplicon of approximately 100 bp is seen. This presumably represents a heteroduplex comprising both amplicons.

**Figure 5:**
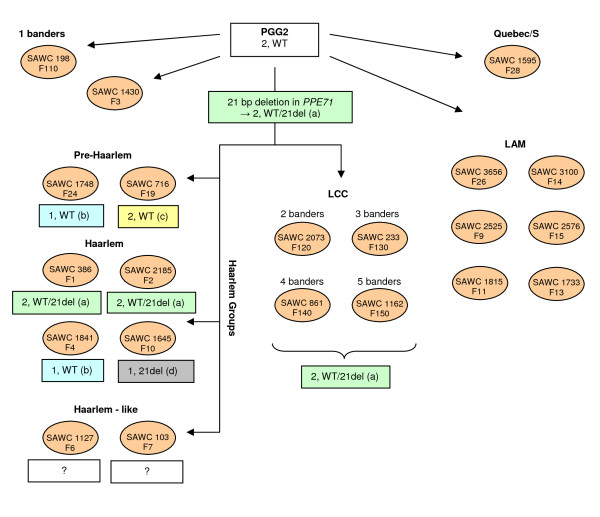
**21del genotype results in relation to PGG2 phylogeny**. A simplified phylogenetic tree of PGG2 lineages shows that the 21del mutation is seen only within the Haarlem and LCC lineages indicating that they share a recent common ancestor. Results also suggest frequent gene conversion and recombination events, particularly between the Haarlem groups. Each mutational type is shown in colour and indicates the number of *PPE38/71 *genes present and the genotype (WT = wildtype, ie lacking the 21del mutation). Green (a): 21 bp deletion (21del) in *PPE71*, both genes retained; Blue (b): Recombination between *PPE38/71 *with *PPE71 *deletion; Yellow (c): Gene conversion leading to deletion of *PPE71 *and duplication of *PPE38*; Grey (d); Recombination between *PPE38/71 *with *PPE38 *deletion. The "Haarlem-like" lineages (F6 and F7) could not be included in the analysis because the 3' end of *PPE38*, including the region homologous to *PPE71 *21del, has been deleted due to an IS*6110*-associated deletion event [see additional file 2, S11 and S12].

The 21del mutation consists of an in-frame 21 bp deletion that results in the loss of amino acids 357 - 63 and was initially identified in *PPE71 *of the CDC1551 whole genome sequence as well as in our clinical isolate SAWC 1645 (Haarlem, F10). The *M. tuberculosis *strain C, which possesses the RvD7 deletion, also shows this mutation, demonstrating that *PPE38*, rather than *PPE71*, has been deleted in this case. Interestingly, while all are PGG2 members, SAWC 1645 belongs to F10 of the Haarlem lineage while CDC1551 and strain C belong to the LCC lineage ("4 banders" and "3 banders" respectively). In order to further track the presence of the 21del mutation in our clinical isolates, PCR primers were designed to distinguish between the 21del (90 bp) and wild type (111 bp) genotypes (Table 1). This PCR was initially performed on all PGG2 isolates from our cohort. Results revealed the presence of this mutation in all 4 members of the LCC ("2, 3, 4 and 5 banders") as well as in 5 of 8 lineages representing the Haarlem, Pre-Haarlem and Haarlem-like clades (Figure 4). A simplified phylogenetic tree of PGG2 lineages in relation to their 21del genotypes is shown in Figure 5. All LCC members showed a "heterozygote-like" "2, WT/21del" (2 genes, 1 wild type, 1 21del) genotype, suggesting the CDC1551-like structure, while results for the Haarlem lineages were more variable with "homozygote-like" signals for both the wild type and 21del genotype observed (Figures 4 and 5). In these cases PPE38F/R and PPE38IntF/IntR PCRs were used to determine the number of *PPE38/71 *genes present and thus distinguish between recombination (1 gene) and gene conversion (2 genes) events. Figure 5 shows that recombination and gene conversion events were observed within the F1, 2, 4, 10, 19 and 24 Haarlem lineages. Mutational analysis of the 2 Haarlem-like lineages (F6 and F7) suggests that the 3' region of *PPE38*, including the region corresponding to the 21del position in *PPE71*, has been removed by an IS*6110*-associated mutation [see additional file 2, S11 and S12]. The "1, 21del" genotype seen in both these lineages is therefore not due to recombination with deletion of *PPE38*.

We next performed the 21del PCR on additional isolates from the various LCC and Haarlem lineages in order to determine whether the observed genotypes were cluster or lineage-specific and also to investigate any additional instances of gene conversion or recombination. Four LCC "6 bander" isolates, which represent a lineage not originally used in our study, were also included. Results are shown in Table 2 and show that 90 additional isolates (including the Haarlem F4, CDC1551 and strain C whole genome sequences), representing 5 LCC and 8 Haarlem lineages (including Haarlem, Pre-Haarlem and Haarlem-like lineages) were analysed. Of these, 5 isolates (5.6%) displayed an altered genotype compared to the standard genotype observed within their lineage. Where genotypic changes were observed the PPE38F/R and PPE38IntF/IntR PCRs were again used to differentiate between recombination and gene conversion events (Table 2) No cases of within-cluster genotypic alterations were observed.

**Table 2:** 21del analysis of lineages representing the LCC and Haarlem groups

**Group/Lineage**	**Same cluster**	**Different clusters**	**Total**	**Standard genotype**	**Mutations observed**
LCC, 2 banders	4	N.A.	4	2, WT/21del	0
LCC, 3 banders	6	N.A.	6	2, WT/21del	1. (1, 21del)
LCC, 4 banders	6	N.A.	6	2, WT/21del	0
LCC, 5 banders	6	N.A.	6	2, WT/21del	1. (1, WT)
LCC, 6 banders	4	N.A.	4	2, WT/21del	0
F6. Haarlem-like^†^	3	3	6	1, 21del	0
F7. Haarlem-like^†^	3	5	8	1, 21del	0
F1. Haarlem	-	3	3	2, WT/21del	0
F2. Haarlem	5	8	13	2, WT/21del	1. (1, 21del)
F4. Haarlem	4	6	10	1, WT	0
F10. Haarlem	4	2	6	1, 21del	0
F24, Pre-Haarlem	4	5	9	1, WT	2. (2, WT/21del)
F19, Pre-Haarlem	4	5	9	2, WT	0

**Total**	53	36	90		5

N.A.: Not applicable. Because of the invariance of the IS*6110 *RFLP patterns LCC lineages cannot be subdivided into clusters.

^†^The "1, 21del" genotype observed in these lineages is due to deletion of the 3' end of *PPE38 *by an IS*6110*-mediated mechanism rather than homologous recombination with deletion of *PPE38*.

### Analysis of the *plcABC *genes from publicly available whole genome sequences

Previous reports have revealed that the genomic region adjacent to *PPE38 *encompassing the three phospholipase (*plc*) gene loci *plcA, plcB *and *plcC *is subjected to frequent deletions and IS*6110 *insertions [26-30]. We therefore also examined this region in the 15 publicly available *M. tuberculosis *whole genome sequences and 8 non-*M. tuberculosis *MTBC members described above. Numerous mutations were observed. These included SNPs, micromutations that resulted in frameshifts and altered amino acid incorporation, IS*6110 *integration and a case of gene fusion between *plcA *and *plcB *in isolate 02_1987. Some of the observed SNPs were found to be lineage-specific. For example, a sSNP (A → C) at position 435 of *plcA *distinguished all "ancient" strains (TBD1+) from "modern" strains (TBD1-) [31]. Large RD5 and RD5-like deletions have been previously shown to affect these genes in *M. bovis*, *M. bovis *BCG, [24,25], *M. microti *(where it is part of the RD5^mic ^deletion [32]), the dassie bacillus (where it is part of the RD5^das ^deletion [22]) and in the oryx bacillus (where it is part of the RD5^oryx ^deletion [23]) (Figure 3). Two of the *M. tuberculosis *isolates (T92 and 94_M4241A) showed similar RD5-like deletions (Figure 3). A complete summary of the results can be seen in Table 3.

**Table 3:** Mutational analysis of the *plcABC *genes from 15 publicly available whole genome *M. tuberculosis *isolates and 8 non-*M. tuberculosis *MTBC members.

**Isolate**	***plcA***	***plcB***	***plcC***	**Comment**
*M. bovis*	Deleted	Deleted	Deleted	*plcABC *part of the RD5 region (Figure 3).
*M. bovis *BCG	Deleted	Deleted	Deleted	*plcABC *part of the RD5 region (Figure 3).
CPHL_A	sSNP A → C at position 435.	+	+	All genes predicted to be fully functional.
K85	sSNP A → C at position 435.	+	nsSNP C → T (Thr → Ile) at aa position 302. sSNP G → A at position 1506.	plcC function possibly impaired.
GM041182	sSNP A → C at position 435.	+	+	All genes predicted to be fully functional.
*M. microti*	Deleted.	Deleted	5' 867 bp deleted.	*plcABC *part of the RD5^mic ^region (Figure 3).
Oryx bacillus	Deleted	5' 260 bp deleted.	+^‡^	*plcAB *part of the RD5^oryx ^region (Figure 3).
Dassie bacillus	Deleted	Deleted	Deleted	*plcABC *part of the RD5^das ^region (Figure 3).
T17	sSNP A → C at position 435.	IS*6110 *insertion at position 1307.	+	plcB function predicted to be abolished.
EAS054	sSNP A → C at position 435.	sSNP G → A at position 1404.	+	All genes predicted to be fully functional.
T92	Deleted	Deleted	5' 194 bp deleted.	Major deletion results in removal of *plcA*, *plcB *and 5' region of *plcC *(Figure 3).
94_M4241A	Deleted	5' 793 bp deleted.	sSNP T→ C at position 753.	IS*6110*-associated recombination event has deleted *plcA *and 5' region of *plcB *(Figure 3).
02_1987	Deletion of 3' end of *plcA *and 5' end of *plcB *creates hybrid *plcA/B *gene. Fusion point at position 145. Results in frameshift and premature protein termination.		sSNP T→ C at position 753.	Hybrid *plcA/B *gene predicted to be non-functional. Part of massive genome rearrangements seen in this isolate (Figure S23).
T85	sSNP G → A at position 705. nsSNP T → A (thr → ala) at position 1336.	+	sSNP T → C at position 753. nsSNP G → T (gly → cys) at position 1081.	plcA and plcC functions possibly impaired.
KZN 4207	+	+	+	Total homology to H37Rv.
KZN 1435	+	+	+	Total homology to H37Rv.
KZN 605	+	+	+	Total homology to H37Rv.
F11	+	+	+	Total homology to H37Rv.
Strain C	T insertion at position 104. Altered reading frame and premature protein termination.	+	+	*plcA *function predicted to be abolished.
CDC1551	+	+	+	Total homology to H37Rv.
Haarlem	A insertion at position 968. Altered reading frame and premature protein termination.	+	+	*plcA *function predicted to be impaired.
H37Rv	+	+	+	Defined as wild type sequence
H37Ra	+	+	+	Total homology to H37Rv.

+ indicates complete homology to the H37Rv reference sequence.

^‡^Deletion mapping studies indicates that the *plcC *gene of the Oryx bacilli is present [23]. The exact sequence of the gene in this species is unknown however.

### Analysis of the *PPE39 *and *PPE40 *genes from publicly available whole genome sequences

As described above, many instances of *PPE38*-encompassing RD5-like deletions that span the region from *plcABC *to *PPE39 *or *PPE40 *have been detected in the non-*M. tuberculosis *MTBC members [22-25,32]. Similar deletions were also found in the T92 and 94_M4241A *M. tuberculosis *isolates (Figure 3, Table 4). We analysed the mutational status of the *PPE39 *and *PPE40 *genes in all available whole genome sequences in order to determine the extent of the hypervariable region that appears to be centered around *PPE38*. Our analysis demonstrates that, apart from RD5-like deletions, both genes are frequently subjected to additional mutational events at both the micro- and macro-mutational scale and that in many cases the resultant protein function is predicted to be abolished or altered (Table 4). Several mutations are of particular interest. Isolates 94_M4241A and *M. bovis *BCG both revealed the presence of a single *PPE39*/*40 *fusion gene (Figure 3). Analysis of the DNA sequences of *PPE39 *and *PPE40 *genes shows that they are identical to position 538 (N-terminal conserved region) after which they diverge. The *PPE39/40 *fusion genes possesses *PPE40 *upstream sequence indicating that this portion of the gene is indeed *PPE40*. However, the sequences following position 538 are specific to *PPE39 *suggesting that the fusion has occurred at the point of divergence. The resultant proteins are thus predicted to be identical to *PPE39 *although the upstream regulatory sequences correspond to *PPE40*. Also of note was the finding that, despite being unrelated, the *PPE39 *gene in isolates CDC1551 and EAS054 both share the same 3 bp in-frame deletion of nucleotides. The deletion occurs at a trinucleotide repeat region (4 × GCG, positions 79 - 90) and we predict that microsatellite instability has resulted in independent deletion events to both these isolates. Finally, we also found that the Haarlem and F11 isolates share a direct IS*6110 *integration at position 47 of *PPE39 *with a resultant GGA duplication at the site of insertion. Interestingly, isolate CPHL_A has an identical IS*6110 *insertion in *PPE40 *and isolate 02_1987 also reveals an IS*6110 *integration at this point. A more detailed analysis of this apparent sequence-specific hotspot for IS*6110 *integration has recently been accepted for publication.

**Table 4:** Mutational analysis of the *PPE39 *and *PPE40 *genes from 15 publicly available whole genome *M. tuberculosis *isolates and 8 non-*M. tuberculosis *MTBC members.

**Isolate**	***PPE39***	***PPE40***	**Comment**
*M. bovis*	Deleted downstream from position 1358.	3 bp in-frame deletion removes aa 164 (A).	*PPE39 *part of RD5 region (Figure 3).
*M. bovis *BCG	*PPE39*/40 gene fusion. 3 bp in-frame deletion seen in *M. bovis PPE40 *also present.		Fused *PPE39/40 *(Figure 3).
CPHL_A	+	IS*6110 *integration at position 47.	PPE40 function predicted to be abolished.
K85	sSNP G→ T position 1548	+	
GM041182	sSNP C→ T position 1563.	33bp in-frame deletion of nucleotides 190 -- 222. Removes aa sequence AAAAAMVVAAA.	PPE40 function predicted to be altered.
*M. microti*	Deleted downstream of position 325.	+	*PPE39 *is part of the RD5^mic^region (Figure 3).
Oryx bacillus	Deleted	Deleted	*PPE39 *and *PPE40 *are parts of the RD5^oryx^region (Figure 3).
Dassie bacillus	Deleted	Gene present.	Deletion analysis suggests that *PPE40 *is intact but exact sequence is unknown.
T17	+	+	
EAS054	3 bp (GCG) in-frame deletion removes alanine at aa position 27.	+	
T92	Deleted	Deleted from position 1592.	See Figure 3.
94_M4241A	*PPE39*/40 gene fusion.		Fused *PPE39/40 *(Figure 3).
02_1987	Deleted	IS*6110 *integration at position 47. 3' region of gene deleted.	Most of genes deleted as part of major genomic structural alterations. (Figure S23).
T85	G insertion position 830, A deletion position 942. Stop codon aa position 278.	+	PPE39 function predicted to be abolished or highly modified.
KZN 4207	+	+	
KZN 1435	+	6 nsSNPs between positions 1094 and 1105. 2 aa changes: 367 T→ N and 368 G→ N.	
KZN 605	+	+	
F11	IS*6110 *integration at position 47.	+	PPE39 function predicted to be abolished.
Strain C	N.D.	sSNP T→ C position 969.	Unable to characterise *PPE39 *sequence due to the numerous 'N's'. Full length gene present.
CDC1551	3 bp (GCG) in-frame deletion removes alanine at aa position 27.	+	
Haarlem	IS*6110 *integration at position 47.	+	PPE39 function predicted to be abolished.
H37Rv	IS*6110 *integration at position 20. Following IS *PPE39 *sequence commences at position 821.	+	PPE39 function predicted to be abolished.
H37Ra	As for H37Rv.	+	PPE39 function predicted to be abolished.

+ indicates homology to consensus sequence.

### Analysis of the *PPE38 *gene region in non-tuberculous mycobacterial species

**Figure 6:**
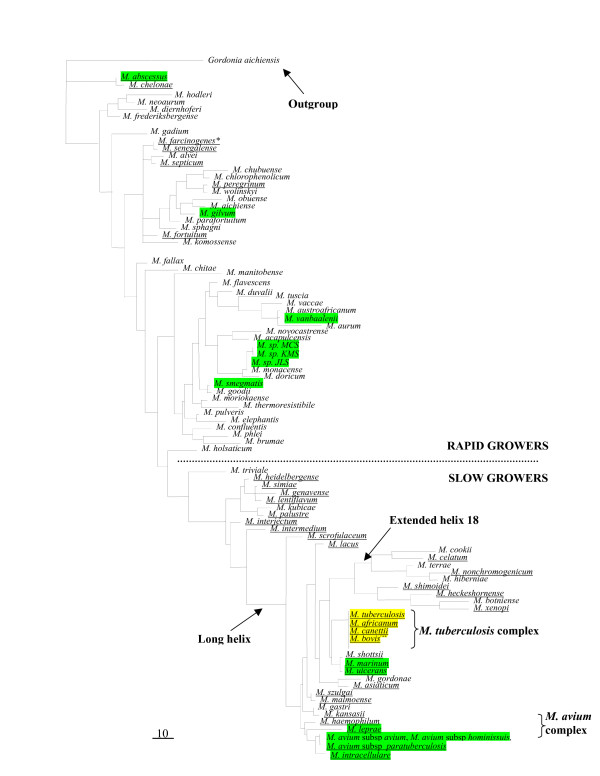
**Phylogeny of Mycobacterial species**. Phylogenetic tree of 80 members of the genus *Mycobacterium *based on the 16S rRNA DNA sequence with the sequence of the species *Gordonia aichiensis *as the outgroup. Reproduced from ref [2] with permission from the authors. MTBC members analysed in this study are highlighted in yellow, while other mycobacteria analysed are highlighted in green.

The extensive variability observed at the *M. tuberculosis PPE38 *region led us to examine its structure in more distant evolutionary time in order to gain insights into its evolutionary history. The whole genome sequences of 7 slow growing and 7 fast growing species of non-tuberculous mycobacteria (Figure 6), as well as several actinobacteria members outside the mycobacterium genus, were analysed for protein sequences showing homology to *PPE38*, MRA_*2374*, MRA_*2375*, *plcABC *and other genes found in the *M. tuberculosis PPE38 *region. The genomic region surrounding proteins of high homology was examined for similarities to the *M. tuberculosis *structure.

**Figure 7:**
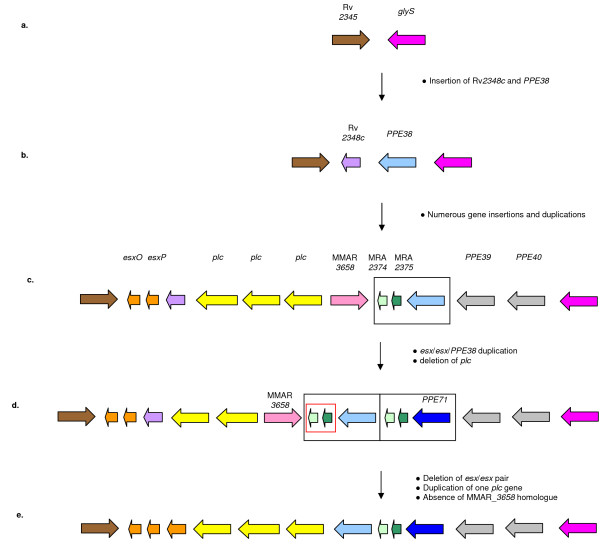
**Possible evolutionary scenario for the MTBC *PPE38 *gene region**. Analysis of fast growing mycobacterial species and the *M. avium *complex indicates that the homologues of Rv2345 and *glyS *have been in close proximity for a long evolutionary period and that the insertion of homologues to *PPE38 *and Rv2348*c *between these genes was also a relatively early event (a, b). The most recent common ancestor of the *M. marinum*/*M. ulcerans *and MTBC lineages is hypothesised to have comprised a single *esx*/*esx*/*PPE38 *gene cluster (black box) located between the *plcABC *(yellow) and *PPE39*/40 (grey) gene regions (c). Duplication of *esx*/*esx*/*PPE38 *resulted in a genotype that is retained by *M. marinum *(d). The genotype of the ancestral MTBC species (e) shows an additional deletion of the *esx*/*esx *gene pair between *plcA *and *PPE38 *(red box).

We first investigated the structure of this region in actinobacteria outside of the genus *Mycobacterium *(including members of the genera *Corynebacterium, Rhodococcus*, and *Nocardia*). In all cases *glyS *and an orthologue of Rv*2345 *(which are both located near *PPE38 *in *M. tuberculosis*, see Figures 3 and 7) could be found situated in close proximity to each other and in all cases they were separated by between 1 and 5 genes. These genes are unrelated to any genes found in the *PPE38 *region in the mycobacteria.

#### The fast-growing mycobacteria - *M. smegmatis, M. sp. JLS, M. sp. MCS, M. sp. KMS, M. vanbaalenii, M. gilvum*, and *M. abscessus*

The *M. smegmatis *genome contains a region homologous to the *M. tuberculosis esxA/esxB *operon found in the RD1 region [33]. However, the single *PE/PPE *and *esx *gene pairs located within this region are the only ones present in the genome and it has previously been demonstrated that *PE/PPE *expansion has only occurred in certain slow growing mycobacteria and not in the fast-growers [2]. A number of fast-growing mycobacterial genomes have been sequenced, including *M. smegmatis, M. sp. JLS, M. sp. MCS, M. sp. KMS, M. vanbaalenii, M. gilvum*, and *M. abscessus*. Analysis of the genomes of these organisms confirmed the absence of any *PE/PPE *genes outside of the *esx*-regions. The *PPE38 *surrounding region was identified in *M. smegmatis, M. sp. JLS, M. sp. MCS, M. sp. KMS *and *M. gilvum *to only contain two genes, namely *glyS *and the orthologue of *Rv2345*. This seems to represent the structure of the region before the insertion of *PPE38 *and the other genes found in this region in the slow-growing mycobacteria (Figure 7). The genome of *M. vanbaalenii *contains the same region with the insertion of an orthologue of Rv*2248 *between *glyS *and the orthologue of Rv*2345*. The other *PPE*, *esx *and *plcABC *genes are absent from the regions and the rest of the genomes of these organisms.

*M. abscessus*, which is one of the earliest mycobacterial species to diverge within the genus *Mycobacterium*, has an expanded region containing *glyS*, an aminotransferase, an 1-aminocyclopropane-1-carboxylate deaminase, an GntR family transcriptional regulator, and the orthologue of Rv*2345*. It is unclear whether the genes between *glyS *and the orthologue of Rv*2345 *have been inserted or whether this represents the ancient structure of the region.

#### *M. avium *complex (*M. avium *subsp. *hominissuis, M. avium *subsp. *avium, M. avium subsp. paratuberculosis *and *M. intracellulare*)

The whole genomes of four members of the *M. avium *complex have been sequenced, namely *M. avium *subsp. *hominissuis*, *M. avium *subsp. *avium*, *M. avium *subsp. *paratuberculosis *and *M. intracellulare*. Analysis of the genomes of these four organisms revealed the presence of a region containing orthologues to the genes found in the *PPE38 *region. However, this region is substantially reduced and only contains orthologues of *glyS*, *PPE38*, Rv*2348c *and Rv*2345*. The other *PPE*, *esx *and *plcABC *genes are absent from the region and the rest of the genome. From this result it seems that the ancestral region only consisted of these four genes (Figure 7).

#### *M. leprae*

A substantially reduced *PPE38 *region was identified in the genome of *M. leprae*, which contains only *glyS*, an IS*6110 *element (pseudogene - ML*0827c*), *PPE38 *(pseudogene, named *PPE7 *in the *M. leprae *database), *plcA *(pseudogene) and the orthologue of Rv*2345 *(pseudogene - ML*0830c*). Due to the extreme reductive evolution of this organism's genome [34], it is unclear what the original structure of this region in *M. leprae *was before genome downsizing, so this organism was also not found to be useful for investigating the evolution of this region.

#### *M. marinum*

*M. marinum *and *M. ulcerans *share a recent common ancestor and both are also closely related to the MTBC (Figure 6). *M. marinum *has the most extensive *PE*/*PPE *gene repertoire yet discovered and contains 105 *PPE *genes [35]. BLAST analysis of *M. marinum *proteins with the *PPE38 *amino acid sequence identified 2 genes (MMAR_*3661 *and MMAR_*3664*) with highest homology. Two *esx*-like genes are located downstream from each *PPE38/71 *homologue suggesting that the *M. tuberculosis PPE38 *region evolved initially by duplication of a *esx/esx/PPE *sequence, to produce the structure seen in *M. marinum*, followed by deletion of the *esx *gene pair downstream of *PPE38 *homologue MMAR_*3661 *(Figure 7). The homology of this region with the *M. tuberculosis PPE38 *region is further confirmed by surrounding genes including the upstream genes MMAR_*3665 *(highest homology to *PPE39*), MMAR_*3666 *(*PPE40*) and MMAR_*3667 *(*glyS*). As in *M. tuberculosis*, the *plc *region is located downstream from MMAR_*3661*. Unlike *M. tuberculosis *the *PPE38/71 *homologues are not identical but show 95% homology at the amino acid level. However, the *esxN 4/esxN 5 *(homologous to MRA_*2374*) and *esxP 4/esxP 5 *(homologous to MRA_*2375*) gene pairs are identical both to each other and to their *M. tuberculosis *counterparts. In the light of these findings we were interested to know whether *M. canettii *(the most ancestral MTBC member) also retained the *esx *gene pair located between *plcA *and *PPE38*. Using the plcA5'/PPE38IntF primer pair (Table 1) we amplified the region between *plcA *and *PPE38 *in our 3 *M. canettii *clinical isolates. Amplicon size indicated the *M. tuberculosis *structure with loss of the 2 *esx *genes observed in *M. marinum *and suggests that the ancestral MTBC organism had this deletion (Figure 7). Sequence analysis of 1 amplicon confirmed that, apart from several intergenic SNPs, the structure was identical to that seen in H37Rv.

#### *M ulcerans*

The genome sequence of *M. ulcerans *shows that it has recently evolved from a *M. marinum*-like ancestor that acquired a virulence plasmid from another actinobacterium [36]. Since their divergence *M. ulcerans *has undergone extensive reductive evolution that has included genome downsizing [37]. This has resulted in alterations to the *PPE38 *region and no region of significant homology could be found. This organism was thus not found to be useful for investigating the evolution of this region.

## Discussion

Using PCR and sequencing-based analysis of clinical isolates in conjunction with data obtained from publicly available whole genome sequences of *M. tuberculosis*, non-*M. tuberculosis *MTBC members and other non-tuberculous mycobacteria, we have investigated alterations of the *PPE38 *gene region, along with its evolutionary history. Analysis of the *M. marinum *whole genome sequence shows that the MTBC *PPE38 *region probably arose from the duplication of an *esx*/*esx*/*PPE38 *gene cluster followed by the deletion of one *esx/esx *gene pair (Figure 7). The more ancient evolution of the region is difficult to interpret from the available mycobacterium genome sequences. Analysis of the *M. avium *complex suggests that the insertion of *PPE38 *between Rv*2345 *and *glyS *was an early event but the exact timing of the *esx *and *plc *gene appearances remains unresolved. These questions will only be answered by the sequencing of more Mycobacterial species evolutionary situated close to the *M. tuberculosis *and *M. avium *complexes (e.g. *M. kansasii*) as well as additional species located on different phylogenetic branches, such as *M. gordonae*/*M. asiaticum *and members of the extended helix 18 group such as *M. terrae *(Figure 6).

Our results demonstrate that the *M. tuberculosis PPE38 *region is hypervariable, adding to mounting evidence indicating that MTBC genomes are not as homogeneous as previously thought [38], and that they have undergone, and continue to undergo, considerable divergence from their most recent common ancestor. From a total of 69 MTBC isolates analysed 36 (52%) were found to contain major structural alterations. When smaller micromutations that are predicted to alter *PPE38 *or *PPE71 *protein function are included in this tally only 22 isolates (32%) remain that show the ancestral H37Ra-like structure (Figure 2b) containing the identical *PPE38 *and *PPE71 *genes. It should be noted that several of the analysed isolates were close relatives (e.g. SAWC 2576, KZN 4207, KZN 1435 and KZN 605 all belong to F15) and thus our mutation frequency may be a slight overestimate. However, countering this is the fact that genotypic analysis for many of our clinical isolates was based on PCR analysis rather than sequencing and additional micro-mutations may have gone undetected.

The hypervariability of the *PPE38 *region results from the combination of a high frequency of IS*6110 *integration events, IS*6110*-associated recombination/deletion events, homologous recombination and gene conversion events. The frequency and variety of IS*6110*-associated mutations observed was striking. At least 20 of the 69 isolates (29%) displayed IS*6110*-associated mutations and these ranged from direct integrations, both into genes and intergenic regions, to recombination events that resulted in partial or full gene deletions. IS*6110 *integrations were also found to be common in *PPE39 *and *PPE40 *(Table 4) and they are also implicated in the large RD5-like deletions observed in isolate 94_M4241A and members of the non-human animal adapted MTBC members (Figure 3). The reason for the high IS*6110 *activity within this, or any of the other previously described *M. tuberculosis *IS*6110 *hotspot regions [39-42], is unclear. The element does not display any obvious insertion site sequence specificity, although in our analysis of *PPE38*, *PPE39 *and *PPE40 *we documented multiple, independent, identical integration sites. A more detailed analysis of this finding has recently been accepted for publication. Also of note is the finding that of all IS*6110 *integrations that were found to disrupt the 4 *PPE *genes analysed here, all occurred in their 5' (conserved N-terminal) regions. Apart from the obvious negative functional effects of gene deletion and disruption, IS*6110 *can also function as a mobile promoter and upregulate genes located downstream of its integration site [43-45]. Three of our clinical isolates revealed IS*6110 *integrations upstream of genes and an investigation into the transcriptional effects could be of interest. The dynamic nature of the genome in this region in relation to IS*6110*-associated integration and recombination is further evidence for the role of IS*6110 *in the generation of genome plasticity in *M. tuberculosis *and its influence on the organism's evolution [46].

The finding that 10 of the 69 isolates harboured the RvD7 genotype demonstrates a high frequency of homologous recombination between *PPE38 *and *PPE71*. Additional analysis of homologous recombination and gene conversion between these genes was greatly aided by the identification of the 21del mutation. This in-frame deletion has allowed us to distinguish between the 2 genes in the PGG2 LCC and Haarlem groups. 21del analysis demonstrated a high frequency of both homologous recombination and gene conversion, particularly between the various Haarlem groups, that result in various combinations of single/double/wildtype/mutant genotypes (Figures 4 and 5, Table 2). Springer and colleagues [47] have reported that in *M. smegmatis *homologous recombination can only originate in regions of high (> 99%) sequence homology but, once initiated, can extend across heterologous regions with limited constraint. Termination of the event was found to require another region of high sequence similarity. This is consistent with the situation seen between *PPE38 *and *PPE71 *either with or without the 21del mutation.

The case for a high frequency of gene conversion between *PPE38 *and *PPE71 *is supported by comparisons of the *M. tuberculosis *and *M. marinum *genomes. We found that within each genome there is extreme homology between the *PPE38/71 *and MMAR_*3661*/MMAR_*3664 *genes (over 95% in *M. marinum *and generally 100% in *M. tuberculosis *at both the DNA and protein level), while between genomes the homology between *PPE38/71 *and MMAR_*3661 *and MMAR_*3664 *is only 86% at the DNA level and 37% and 36% respectively at the protein level. This extreme intra-genomic but lower inter-genomic homology strongly suggests that both pairs of genes have diverged from a recent common ancestral sequence but have been prevented from significant intra-genome divergence by regular gene conversion events. Additional evidence is provided by the sequence of these genes in *M. canettii*. Here, each gene contains a non-synonymous SNP (A → C) at nucleotide position 1054. This indicates that mutation in one gene followed by gene conversion has occurred in either the *M. tuberculosis *or *M. canettii *lineages since they last shared a common ancestor. Gene conversion between *PPE38 *and *PPE71 *could thus explain the apparent paradox between a high macro-mutational frequency, suggesting non-essentiality of the genes, and low micro-mutational frequency, which would normally be an indication of gene essentiality.

These results add to accumulating evidence supporting frequent *PE*/*PPE*-associated homologous recombination and gene conversion in *M. tuberculosis*. Using a microarray-based methodology Karboul and colleagues [48] mapped numerous deletion mutations spanning adjacent *PE *and *PPE *genes and found that they resulted in in-frame fusion genes. Homologous recombination, using the highly conserved N-terminal gene regions as substrates, was strongly implicated in these events. Our own analysis of the *PPE39*/*40 *fusion gene observed in the *M. bovis *BCG and 94_M4241A whole genome sequences provides support for this finding. Two additional reports have provided evidence for between-strain recombination in close proximity to *PE *and *PPE *genes and the authors have proposed the existence of recombination hot spots within or close to these gene family members [49,50]. Regarding gene conversion, the use of the 21del polymorphism to detect this event in 2 highly homologous proximal genes is similar to that recently reported for the *PE_PGRS17 *and *PE_PGRS18 *gene pair [51]. This study reported the presence of a 12 bp insertion associated with a set of 40 SNPs that is found in either *PE_PGRS17 *alone or in both genes. Analysis of this polymorphism in isolates representing a broad spectrum of *M. tuberculosis *lineages shows that numerous gene conversion events have occurred between these genes throughout the evolutionary history of the PGG2 and PGG3 groups. Apart from its utility in detecting *PPE38/71 *recombination and gene conversion events, the 21del mutation is of interest for additional reasons. Firstly, it confirms a close evolutionary relationship between the PGG2 groups, LCC and Haarlem, recently identified by our group (N. C. Gey van Pittius, unpublished results). Secondly, the mutation has become fixed in the majority of lineages and clusters from within these groups, indicating that it might provide the organism with a survival advantage that is able to override the homogenising effect of recombination/gene conversion events. Indeed, this mutation may represent the initial stages of evolutionary divergence between these 2 genes.

Homologous recombination/gene conversion events are also presumably responsible for the indel mutations involving the exchange of *PPE38*/*71 *upstream sequence regions observed in several isolates. Typically, both genes share the same upstream sequence to position - 35 before diverging. The finding that isolate SAWC 3656 contains an indel upstream of *PPE38 *that involves replacement of the normal sequence from position -36 to -83 with *PPE71 *upstream sequence indicates a gene conversion event where *PPE71 *has replaced *PPE38 *in an imperfect recombination that has included a portion of its 5'-untranslated region. The other examples, and particularly 02_1987, indicate that homologous recombination can also produce more complex results. Isolate 02_1987 is a particularly good example of the benefits of whole genome sequence analysis with respect to the large-scale mutational events described in this study. Along with the *plcA/B *and *PPE39/40 *mutations previously described (Tables 3 and 4), this genome was also found to possess numerous additional gene truncations, inversions and IS*6110 *insertions involving both *PPE38*-associated genes and others [see additional file 2, S23] and it provides an idea of the amount of genomic plasticity that can be tolerated by a *M. tuberculosis *isolate that has successfully infected a host and caused disease.

Because our sample cohort is well-defined in terms of evolutionary relationships we were able, in many instances, to determine mutation status at the lineage-, cluster- or isolate-specific level. Although most mutations were found to be lineage-specific, in 5 cases at least one isolate that represented a different cluster from the same lineage revealed an altered genotype. Thus, genotypic variability was often observed within RFLP-defined lineages. Variability was generally not observed within clusters although in most cases the numbers analysed were limited. However, our analysis demonstrated that within cluster alterations can occur with one lineage showing 4 distinct mutations, including 3 within the same cluster [see additional file 2, S7]. These results emphasise the hypervariability of the *PPE38 *region and demonstrate its rapid ongoing evolution at the within-lineage and even the within-cluster level.

Our results show that the *PPE38 *region's hypervariability extends to the adjacent *plcABC *and *PPE39*/*40 *regions. The *plcABC *region has previously been reported as a preferential region for IS*6110 *integration [29] and our results thus extend this region from *plcC *to *PPE40*. This results in a "hot-spot region" of around 11.3 kb when using the CDC1551 sequence as a reference. The importance of the *plcABC *genes, along with *plcD*, which is located in another genomic region, has been emphasised by knockout experiments showing that triple (*plcABC*) or quadruple knockouts are impaired during the late phase of infection in a mouse model [52]. However, several examples of clinical isolates that possess mutations in all 4 *plc *genes have been reported [28,30], revealing that their functions are not always essential for the bacteria's pathogenicity. The finding that the *plcABC *region is deleted in many non-*M. tuberculosis *MTBC members [22-25,32], is further evidence for their limited phenotypic impact (at least in their non-human hosts). Our analysis revealed large scale mutations (deletions or IS*6110 *insertions) in 22 of a potential 69 (23 × 3) *plcABC *genes analysed and indicated that 5 isolates (*M. bovis*, *M. bovis *(BCG), *M. microti*, Dassie bacillus and T92) had functional loss of all 3 *plcABC *genes (Table 3). This mutation frequency is around double that found in the extensive study of Kong and colleagues [30]. This difference might reflect the greater accuracy of whole genome sequence analysis compared to a methodological approach based on PCR and Southern analysis, along with the fact that we included non-*M. tuberculosis *MTBC members with known RD5 or RD5-like deletions in our analysis. Several other micromutations (nsSNPs and microinsertions) were also detected that are predicted to abolish or alter protein function (Table 3). These results confirm the frequent loss of function of these genes in clinical isolates and suggest that previous studies may have underestimated this frequency.

The susceptibility of this region to large deletions is emphasised from analysis of other MTBC members where similar, yet distinct, deletions, which may include adjacent *plc *and *PPE39 *and *PPE40 *genes (RD5 and RD5-like deletions), have been reported in *M. bovis*, *M. bovis *(BCG), *M. caprae*, *M. microti*, and the dassie and oryx bacilli [22-25,32] (Figure 3). RD5-like deletions were found to be less common in *M. tuberculosis *isolates and were observed in just 1 of our clinical isolates and 2 of the *M. tuberculosis *whole genome sequences (Figure 3). The relatively low frequency of RD5-like deletions in *M. tuberculosis *is supported by the findings of Tsolaki and colleagues [53] who identified only 1 such deletion in a total of 100 phylogenetically diverse strains. This low deletion frequency in *M. tuberculosis *compared to non-*M. tuberculosis *MTBC members may signify that the absence of this region may provide the organism with a selective advantage in non-human hosts, a hypothesis that is strengthened by the finding that the RD5^mic ^deletion is found in vole, but not human, *M. microti *isolates [32].

Surprisingly, our analysis of 3 independent H37Rv samples confirmed the typical H37Ra-like structure [19], thus contradicting the published sequence [1] from which the RvD7 genotype is defined. We suggest that the hypervariability of this region may have influenced the results of the published H37Rv whole genome sequence and conclude that the results for this genomic region are not representative of its true sequence. We propose that either a culture-specific *PPE38*/*71 *recombination/deletion occurred to produce the non-representative RvD7 genotype or, alternatively, some subclones used for the H37Rv sequencing project may have become mixed with those from other isolates. The second possibility is supported by analysis of the H37Ra sequence which, surprisingly, was found to be far more similar to the CDC1551 sequence than to H37Rv [19,20]. Whatever the explanation, we suggest that the sequence accuracy of this region for other whole genome sequences that show the RvD7 genotype be treated with caution.

The biological consequences of the described mutations are unknown but our results suggest that functional loss of *PPE38/71*, MRA_*2374 *and MRA_*2375 *(and possibly also *plcABC*, *PPE39*, *PPE40 *or combinations of all of these) do not result in a significant loss of bacterial virulence. We base this conclusion on the high frequency of independent mutations found in this region and the fact that the large number of mutations identified (at least in relation to *PPE38/71*, MRA_*2374 *and MRA_*2375*) were mostly lineage specific, indicating that the original mutated organism had successfully caused disease, transmitted to new hosts and undergone further evolutionary expansion and divergence. The best example of this is that of the typical Beijing's (F29) where IS*6110*-associated recombination/deletion events have resulted in the complete loss of functional *PPE38*, *PPE71*, MRA_*2374 *and MRA_*2375 *[see additional file 2, S4]. Despite this, Beijing F29 represents the dominant *M. tuberculosis *lineage throughout much of Asia and its incidence continues to rise rapidly in many countries and regions throughout the world [54,55]. Beijing F29 is also known to have diverged into many distinct sub-lineages [56,57]. The apparent absence of a deleterious phenotypic effect from mutations in the *PPE38 *region is supported by the transposon site hybridisation studies of Sassetti and colleagues who found that none of the genes analysed in our study were essential for growth either *in vitro *[58] or in an *in vivo *mouse model of infection [59]. In addition to these studies, which relate to *M. tuberculosis *in growth phase, these genes also do not undergo significant differential regulation during dormancy phase [60,61]. The *plc*, *PPE *and *esx *genes are all members of multi-gene families with numerous members within the *M. tuberculosis *genome and it is possible that genetic redundancy is responsible for the observations of Sassetti *et al*. Whether the loss of expression of these genes can, in some cases, be beneficial to the organism remains unclear but many examples of potential "virulence suppressor" genes have been documented [62]. *PlcA*, *PPE *and *esx *genes have all been shown to produce antigenic proteins [7-9,63-65] and it is conceivable that the loss of such potentially potent antigens could aid in immune escape. A recent study [66] has characterised the cellular immune response to 167 peptides representing 8 ORF's (Rv*2346c *- Rv*2353c*) within the RD5 region (referred to in this study by the Behr *et al *[24] annotation, RD7) that is absent in *M. bovis*, *M. caprae *and *M. bovis *BCG compared to *M. tuberculosis*. A high secretion ratio of IFN-γ to IL-10 was observed in response to this peptide pool suggesting that expression of genes within RD5 might produce a protective effect. Loss of genes within this region could therefore result in increased pathogenesis and disease virulence. Finally, our work provides a cautionary note regarding vaccine development studies (which often utilise PE and PPE proteins or peptides) by indicating that at least some PPE gene family members are able to undergo rapid evolutionary change.

## Conclusion

This study presents a detailed analysis of mutations at the *PPE38 *genomic region in a variety of *M. tuberculosis *isolates representing all major evolutionary lineages, along with analysis of this region from other MTBC and non-tubercule mycobacterial species, in order to ascertain its evolutionary history. We conclude that this region is hypervariable due to frequent IS*6110 *integrations, IS*6110*-associated recombination/deletion events, and gene conversion and recombination between *PPE38 *and *PPE71*. Gene conversion was implicated in the low levels of variation observed at the micro-mutational scale between *PPE38 *and *PPE71*. Furthermore, mutational analysis of numerous additional isolates at the lineage and cluster levels has provided insights into the molecular evolution of this region. We describe multiple instances of fixation of *PPE38*-associated mutations at the lineage level, along with examples of within-lineage and even within-cluster variation, indicating rapid and extensive evolution of the region. Because these mutations generally result in the functional loss of genes we conclude that they do not result in a significant loss of fitness and that, since they have been shown to be highly antigenic, they may in fact aid in the organism's survival.

## Methods

### DNA sample collection and determination of strain/lineage/cluster

*M. tuberculosis *isolates from patients residing in an epidemiological field site near Cape Town, South Africa, were genotyped according to the internationally standardized IS*6110 *DNA fingerprinting method [67]. DNA fingerprints were analyzed with GelCompar software, using the unweighted-pair group method using average linkages and Dice coefficients [68]. Isolates with an IS*6110 *similarity index of ≥ 65% were grouped into strain lineages [69]. Spoligotyping was also done to further classify lineages into clades [70].

### PCR and sequencing

All primer sequences are listed in Table 1. PCRs using the PPE38F/R and PPE38IntF/IntR primer pairs were done in a reaction mixture containing 0.1 μg template DNA, 3 μl GC-rich solution, 1.5 μl 10× buffer containing MgCl_2_, 2.4 μl 10 mM dNTP's, 0.6 μl each primer (50 pmol/μl) and 0.12 μl FastStart Taq (Roche, Germany) made up to 15 μl with H_2_O. Amplification comprised an initial 6 min template denaturation followed by 35 cycles of 94°C for 30 s, 57°C 30 s and 72°C 2 min. After the final cycle samples were incubated at 72°C for 7 min. For the 21del analysis samples were subjected to PCR amplification in a reaction mixture containing 0.1 μg DNA template, 1.5 μl 10 × Buffer, 1.2 μl 25 mM MgCl_2_, 2.4 μl 10 mM dNTP's, 0.6 μl of each primer (50 pmol/μl), 0.075 μl HotStarTaq DNA polymerase (Qiagen, Germany) and made up to 15 μl with H_2_O. Amplification was initiated by incubation at 95°C for 15 min, followed by 35 cycles of 94°C for 30 s, 55°C 30 s and 72°C for 5 s. After the final cycle, the samples were incubated at 72°C for 7 min. For sequencing analysis PCR product was electrophoresed through a 1.5% low melting point agarose gel. The amplicon was then cut from the gel and purified using a Promega Wizard SV Gel and PCR Clean-up System (Madison, USA). Sequencing was performed using an ABI 3100 automated DNA sequencer.

### *In silico *whole genome sequence analysis

The following *M. tuberculosis *and *M. africanum *whole genome sequences are available from the Broad Institute Microbial Sequencing Center Databases [71]: *M. tuberculosis *C strain, Haarlem, F11, KZN 4207, KZN 1435, KZN 605, 02_1987, T85, T92, T17, 94_M4241A, EAS054, CPHL_A and K85. The CDC1551 whole genome sequence is available at The Institute for Genomics Research (TIGR) [72]. Analysis of the H37Rv whole genome sequence was performed using the TubercuList website [73]. Analysis of the H37Ra genome along with *M. avium *strain 104, *M. avium *subspecies *paratuberculosis *strain K-10, *M. intracellulare *strain 13950, *M. smegmatis *strain MC2155, *M. abscessus*, *M. gilvum *strain PYR-GCK, *M. sp. JLS*, *M. sp. MCS*, *M. sp. KMS*, *M. vanbaalenii *strain PYR-1, *Nocardia farcinica *strain IFM-10152, *Rhodococcus jostii *strain RHA 1, *Rhodococcus erythropolis *strain PR4, *Corynebacterium glutamicum *strain 13032 and *Corynebacterium diphtheria *strain NCTC 13129 was done using the NCBI genomic BLAST website [74]. Whole genome analysis of other bacterium species was done using the following websites: *M. bovis *strain AF2122/97 - BoviList [75], *M. bovis *BCG strain Pasteur 1173P2 - BCGList [76], *M. africanum *strain GM041182 and *M. microti *strain OV254 - Sanger Centre [77], *M. marinum *strain M - MarinoList [78], *M. ulcerains *strain Agy99 - BuruList [79], *M. leprae *strain TN - Leproma [80], Gene sequence alignments were performed using the CLUSTALW multiple sequence alignment programme [81].

## Abbreviations

CAS: Central Asian clade; EAI: East African-Indian clade; ESAT-6: 6 kDa Early Secreted Antigenic Target (*esx*); F: family/lineage; indel: insertion/deletion where one DNA segment has been deleted and replaced by another; LAM: Latin American and Mediterranean clade; LCC: Low IS*6110 *copy clade; MTBC: *Mycobacterium tuberculosis *complex; PE: protein family characterised by Proline-Glutamic Acid motif; PGG: principle genetic group; PPE: protein family characterised by Proline-Proline-Glutamic Acid motif; PGRS: "polymorphic GC-rich repetitive sequence" subfamily of the PE family; SAWC: South African Western Cape.

## Authors' contributions

CREM, NCGvP, PDvH and RMW conceived and designed the study. CREM carried out all PCR and sequence analysis. CREM and NCGvP carried out bioinformatic analysis. CREM, NCGvP, PDvH and RMW carried out interpretation of the data. CREM drafted the manuscript with assistance from NCGvP, RMW and PDvH. All authors read and approved the final manuscript.

## Supplementary Material

Additional file 1 **Tabulated results of *PPE38 *region analysis**. Summary of the *PPE38 *region genetic structures seen in all 69 samples analysed in this study. Click here for file

Additional file 2 **Detailed structures of variable *PPE38 *regions**. All isolates, including those from whole genome sequence analysis, that did not display the ancestral H37Ra-like genotype are described and, where appropriate, a figure is included below the text. Click here for file
