# Supplementary material for: Evidence for a rapid rate of molecular evolution at the hypervariable and immunogenic Mycobacterium tuberculosis PPE38 gene region
Source: BMC Evol Biol. 2009 Sep 21;9:237. doi: 10.1186/1471-2148-9-237 (PMC2758852; doi:10.1186/1471-2148-9-237)
Supplement: Additional file 1 — Tabulated results of PPE38 region analysis. Summary of the PPE38 region genetic structures seen in all 69 samples analysed in this study. [file 1471-2148-9-237-S1.DOC]

**Additional file 1.** **Summary of the *PPE38* region genetic structures seen in all 69 samples analysed in this study.**

More detailed information of *PPE38* region mutations can be found in the additional file 2 information indicated in the comments column.

**^‡^**Intact genes implies that no macromutations are present.

^†^Genotype determined by whole genome sequence analysis.

*Both intact copies correspond to *PPE71* [see additional file 2, S23].

| **Isolate** | **Principal Genetic Group** | **Clade** | **South African IS*6110* Lineage** | **Intact *PPE38/71* Gene Copies^‡^** | **Comments** | **Reference** |
| --- | --- | --- | --- | --- | --- | --- |
| *M. canettii*.1 | PGG1, TBD1+ | Ancestral MTBC | N.A. | 2 | Full sequencing of the region performed. Two SNPs in *PPE71* and 1 in *PPE38* compared to *M. tuberculosis* sequence. |  |
| *M. canettii*.2 | PGG1, TBD1+ | Ancestral MTBC | N.A. | 2 |  |  |
| *M. canettii*.3 | PGG1, TBD1+ | Ancestral MTBC | N.A. | 2 |  |  |
| *M. bovis*^†^ | PGG1, TBD1+ | MTBC | N.A. | 0 | *PPE38/71* within the RD5 region deleted in *M. bovis* (Fig 3 and additional file 2, S27). | [75] |
| *M. bovis* BCG^†^ | PGG1, TBD1+ | MTBC | N.A. | 0 | *PPE38/71* within the RD5 region deleted in *M. bovis* (Fig 3 and additional file 2, S28). | [76] |
| CPHL_A  (*M. africanum*) ^†^ | PGG1, TBD1+ | MTBC, WA-1 lineage, subtype 1b, sublineage 2 | N.A. | 1 | RvD7 genotype (additional file 2, S32). | [71] |
| K85  (*M. africanum*) ^†^ | PGG1, TBD1+ | MTBC, WA-2 lineage, subtype 1a, sublineage 2 | N.A. | 2 | 6 bp deletion in *PPE38*. Results in incorrect amino acids from position 352 and premature termination (additional file 2, S32). | [71] |
| GM041182 (*M. africanum*^†^ | PGG1, TBD1+ | MTBC, WA-2 lineage, subtype 1a, sublineage 3 | N.A. | 2 | (additional file 2, S32) | [77] |
| *M. microti*^†^ | PGG1, TBD1+ | MTBC | N.A. | 0 | *PPE38/71* within the RD5^mic^ region deleted in *M. microti* (Fig 3 and additional file 2, S29). | [77] |
| Oryx bacillus | PGG1, TBD1+ | MTBC | N.A. | 0 | *PPE38/71* within the RD5^oryx^ region deleted in Oryx bacillus (Fig 3 and additional file 2, S30). | [23] |
| Dassie bacillus | PGG1, TBD1+ | MTBC | N.A. | 0 | *PPE38/71* within the RD5^das^ region deleted in Dassie bacillus (Fig 3 and additional file 2, S31). | [22] |
| SAWC1659 | PGG1, TBD1+ | EAI | N.A. | 2 |  |  |
| SAWC 2493 | PGG1, TBD1+ | EAI | N.A. | 2 |  |  |
| SAWC 4981 | PGG1, TBD1+ | EAI | N.A. | 2 |  |  |
| T17^†^ | PGG1, TBD1+ | EAI | N.A. | 1 | RvD7 genotype (additional file 2, S19). | [71] |
| EAS054^†^ | PGG1, TBD1+ | EAI | N.A. | 1 | RvD7 genotype (additional file 2, S20). | [71] |
| T92^†^ | PGG1, TBD1+ | EAI | N.A. | 0 | RD5-like deletion encompassing entire *PPE38*/*71* region (Fig 3 and additional file 2, S21). | [71] |
| SAWC 2803 | PGG1 | CAS | F34 | 2 |  |  |
| SAWC 2240 | PGG1 | CAS | F20 | 1 | RvD7 genotype. Fully sequenced (additional file 2, S1). |  |
| SAWC 2666 | PGG1 | CAS | F33 | 2 | Full sequencing of the region performed. |  |
| SAWC 974 | PGG1 | CAS | F25 | 2 | Full sequencing of the region performed. |  |
| 94_M4241A^†^ | PGG1 | atypical Beijing (Fig 8) | Pre-F31, 27 | 0 | RD5-like deletion encompassing entire *PPE38*/*71* region (Fig 3 and additional file 2, S22). | [71] |
| 02_1987^†^ | PGG1 | atypical Beijing (Fig 8) | Pre-F31, 27 | 2* | Major genomic rearrangements observed (additional file 2, S23). | [71] |
| SAWC 2088 | PGG1 | atypical Beijing (Fig 8) | F31 | 1 | Region contains mutation involving IS*6110* and insertion/duplication of *PPE71* 5’-untranslated region. Mutation deletes 5’ region of *PPE38* (additional file 2, S2). |  |
| SAWC 2701 | PGG1 | atypical Beijing (Fig 8) | F27 | 0 | IS*6110*-associated recombination event has deleted MRA_*2374*, MRA_*2375* and parts of both *PPE38* and *PPE71* (additional file 2, S3). |  |
| SAWC 2076 | PGG1 | typical Beijing (Fig 8) | F29 | 0 | Identical structure to isolate 2701 except that IS*6110* is in the reverse orientation (additional file 2, S4). |  |
| T85^†^ | PGG1 | typical Beijing (Fig 8) | F29 | 0 | Whole genome sequence incomplete but suggests identical structure to SAWC 2076 (additional file 2, S24). | [71] |
| SAWC 1430 | PGG2 |  | F3 | 2 |  |  |
| SAWC 3656 | PGG2 | LAM | F26 | 2 | Indel mutation in 5’-untranslated region of *PPE38*. Fully sequenced (additional file 2, S5). |  |
| SAWC 2576 | PGG2 | LAM | F15 | 2 | Mutation involving IS*6110* and Indel of *PPE71* 5’-untranslated region between *PPE38* and MRA_*2375* (additional file 2, S6). |  |
| KZN 4207^†^ | PGG2 | LAM | F15 | 2 | Same mutation as SAWC 2576. Single nucleotide insertion in *PPE38* predicted to abolish protein function (additional file 2, S6). | [71] |
| KZN 1435^†^ | PGG2 | LAM | F15 | 2 | Same mutation as SAWC 2576. | [71] |
| KZN 605^†^ | PGG2 | LAM | F15 | 2 | Same mutation as SAWC 2576. | [71] |
| SAWC 2525 | PGG2 | LAM | F9 | 2 |  |  |
| SAWC 1815 | PGG2 | LAM | F11 | 1 | IS*6110*-associated recombination event has removed 3’ region of *PPE71* plus MRA_*2374* and MRA_*2375*. *PPE38* intact. Results confirmed by analysis of F11 whole genome sequence (additional file 2, S7). |  |
| F11^†^ | PGG2 | LAM | F11 | 1 | Same mutation as SAWC 1815. | [71] |
| SAWC 1733 | PGG2 | LAM | F13 | 2 |  |  |
| SAWC 3100 | PGG2 | LAM | F14 | 0 | PPE38F/R, PPE38IntF/R and 21del PCRs fail to produce product suggesting complete deletion of *PPE38/71* region (additional file 2, S8). |  |
| SAWC 1595 | PGG2 | Quebec/S | F28 | 1 | RvD7 genotype. Fully sequenced (additional file 2, S9). |  |
| SAWC 198 | PGG2 | “1 bander” | F110 | 2 |  |  |
| SAWC 2073 | PGG2 | LCC - “2 bander” | F120 | 2 | 21del mutation in *PPE71.* |  |
| SAWC 233 | PGG2 | LCC - “3 bander” | F130 | 2 | 21del mutation in *PPE71.* |  |
| Strain C^†^ | PGG2 | LCC - “3 bander” | F130 | 1 | RvD7 genotype. 21del mutation reveals loss of *PPE38* (additional file 2, S25). | [71] |
| SAWC 861 | PGG2 | LCC - “4 bander” | F140 | 2 | 21del mutation in *PPE71.* |  |
| CDC1551^†^ | PGG2 | LCC - “4 bander” | F140 | 2 | 21del mutation in *PPE71* (additional file 2, S26)*.* | [72] |
| SAWC 1162 | PGG2 | LCC - “5 bander” | F150 | 2 | 21del mutation in *PPE71.* |  |
| SAWC 716 | PGG2 | Pre-Haarlem | F19 | 2 |  |  |
| SAWC 1748 | PGG2 | Pre-Haarlem | F24 | 1 | RvD7 genotype. Fully sequenced (additional file 2, S10). |  |
| SAWC 1127 | PGG2 | Haarlem-like | F6 | 1 | 21del mutation in *PPE71*. IS*6110*-associated deletion of the 3’ end of *PPE38* (additional file 2, S11)*.* |  |
| SAWC 103 | PGG2 | Haarlem-like | F7 | 1 | 21del mutation in *PPE71.* Probable IS*6110*-associated deletion of 3’ end of *PPE38* (additional file 2, S12)*.* |  |
| SAWC 386 | PGG2 | Haarlem | F1 | 2 |  |  |
| SAWC 1645 | PGG2 | Haarlem | F10 | 1? | 21del mutation in *PPE71*. Unable to fully characterise but PCRs suggest 1 intact *PPE71* gene (additional file 2, S13). |  |
| SAWC 1841 | PGG2 | Haarlem | F4 | 1 | RvD7 genotype. Fully sequenced (additional file 2, S14). |  |
| Haarlem^†^ | PGG2 | Haarlem | F4 | 1 | RvD7 genotype. Whole genome sequence analysis (additional file 2, S14). | [71] |
| SAWC 2185 | PGG2 | Haarlem | F2 | 1 | *PPE38* disrupted by IS*6110*. 21del mutation in *PPE71* (additional file 2, S15)*.* |  |
| SAWC 239 | PGG3 | T | F22 | 2 |  |  |
| SAWC 2901 | PGG3 | T | F16 | 2? | Unable to fully characterize. Intergenic IS*6110* insertion between *MRA_2375* and *PPE71* (additional file 2, S16). |  |
| SAWC 1608 | PGG3 | T | F5 | 2 | MRA_*2374* disrupted by IS*6110* (additional file 2, S17)*.* |  |
| SAWC 1109 | PGG3 | T | F23 | 2 |  |  |
| SAWC 1870 | PGG3 | T | F18 | 2 | Full sequencing of the region performed. nsSNP in *PPE71* predicted to abolish protein function. |  |
| SAWC 1956 | PGG3 | T | F17 | 1 | *PPE38* disrupted by IS*6110* (additional file 2, S18)*.* |  |
| SAWC 1290 | PGG3 | T | F21 | 2 |  |  |
| SAWC 300 | PGG3 | T | F12 | 2 | Full sequencing of the region performed. |  |
| SAWC 4302 | PGG3 | T | F8 | 2 |  |  |
| H37Rv^†^ | PGG3 |  | N.A. | 1 | Defined as RvD7 genotype. (Fig 2a) | [73] |
| H37Rv.1 | PGG3 |  | N.A. | 2 | Full sequencing of the region performed. |  |
| H37Rv.2 | PGG3 |  | N.A. | 2 |  |  |
| H37Rv.3 | PGG3 |  | N.A. | 2 |  |  |
| H37Ra^†^ | PGG3 |  | N.A. | 2 | Ancestral MTBC structure (Fig 2b) | [74] |
