# Supplementary material for: Evidence for a rapid rate of molecular evolution at the hypervariable and immunogenic Mycobacterium tuberculosis PPE38 gene region
Source: BMC Evol Biol. 2009 Sep 21;9:237. doi: 10.1186/1471-2148-9-237 (PMC2758852; doi:10.1186/1471-2148-9-237)
Supplement: Additional file 2 — Detailed structures of variable PPE38 regions. All isolates, including those from whole genome sequence analysis, that did not display the ancestral H37Ra-like genotype are described and, where appropriate, a figure is included below the text. [file 1471-2148-9-237-S2.DOC]

**Additional file 2.**

All isolates, including those from whole genome sequence analysis, that did not display the ancestral H37Ra-like genotype (Fig 2b) are described and, where appropriate, a figure is included below the text. Colour coding as follows: *PPE38* pale blue, *PPE71* dark blue, MRA_*2374* pale green, MRA_*2375* dark green, IS*6110* red.

**S1. SAWC 2240 (PGG1, CAS, F20)**

The PPE38F/R PCR produced an amplicon representing the RvD7 genotype (Fig 2a). This was confirmed by sequencing the amplicon. A PCR using the PPE38IntF/IntR primers failed to produce a product, further confirming an RvD7 genotype.

**S2. SAWC 2088 (atypical Beijing, F31)**

Analysis showed an IS*6110* integration that removed the first 50 bp of *PPE38*. In addition, directly adjacent and upstream of the IS element, a 77 bp insertion was observed that was found to be identical to the upstream region of *PPE71* (positions -18 to -95). Normal *PPE38* 5’-untranslated sequence starts at position -84. No duplications of the target sequence at the IS*6110* integration point were observed, suggesting an IS-mediated recombination event. This result was confirmed by sequence analysis of an additional F31 isolate. Furthermore, 8 additional F31 isolates representing 5 distinct IS*6110* RFLP clusters were analysed using the PPE38F/R PCR. In each case the amplicon size was the same as SAWC 2088 suggesting that this genomic structure is F31-specific.

**S3. SAWC 2701 (atypical Beijing, F27)**

Sequence analysis of this isolate revealed the same IS*6110*/*PPE38* junction as described above for the atypical Beijing F31 sample. This is not unexpected as both F27 and F31 have evolved from a recent common ancestor (See figure S22). However, a further recombination event that has led to the deletion of the 3’ region of *PPE71*, along with MRA_*2374* and MRA_*2375*, was also observed. The resultant genomic structure comprises an IS*6110* element flanked upstream by the first 72 bp of *PPE71* and downstream by *PPE38* minus the first 50 bp. This structure was confirmed by sequence analysis of an additional F27 isolate. In addition, 5 more F27 isolates (3 from the same cluster as SAWC 2701 and 2 from different clusters) were analysed by PPE38F/R PCR. In each case the amplicon size was the same as SAWC 2701 suggesting that this genomic structure is F27-specific.

**S4. SAWC 2076 (typical Beijing, F29) and T85 (whole genome sequence)**

The genomic structure of the *PPE38/71* region was found to be almost identical to the atypical Beijing F27 except that the IS*6110* element was orientated in the reverse direction and that it was inserted at *PPE71* position 77 rather than position 72. Lineages 27 and 29 have evolved from a recent common ancestor (see figure S22) suggesting that the IS*6110* element has flipped in its position, or that the IS*6110* element was displaced by the insertion of a new element in the reverse orientation. Eight additional F29 isolates representing 8 different clusters were analysed using the PPE38F/R PCR. In each case the amplicon size was the same as SAWC 2076 suggesting that this genomic structure is F29-specific.

**S5. SAWC 3656 (LAM, F26)**

The PPE38F/R PCR produced an amplicon representing the RvD7 genotype. This was confirmed by sequencing the amplicon. However, a PCR using the PPE38IntF/IntR primers produced the 1351 bp amplicon indicating the typical H37Ra-like genotype. Sequence analysis of this amplicon revealed an indel mutation in the 5’-untranslated region of *PPE38*. This involved a 48 bp deletion from positions -36 to -83 and its replacement with a 70 bp sequence corresponding to the 5’-untranslated region of *PPE71* (positions -34 to -103, black squares below). This insertion contains the PPE38F primer binding sequence and its duplication explains why the H37Ra-like *PPE38/71* genomic structure produced an amplicon representing the RvD7 genotype when amplified with the PPE38F/R primer pair.

**S6. SAWC 2576 (LAM, F15) and KZN 4207, KZN 1435 and KZN 605 (whole genome sequences)**

Our isolate SAWC 2576 as well as the whole genome sequences KZN 4207, KZN 1435 and KZN 605 all belong to PGG2, LAM, F15. The three whole genome sequences derive from closely related F15 members and represent drug susceptible, multi drug resistant and extremely drug resistant isolates. Sequence analysis of SAWC2576 revealed the presence of intact copies of *PPE38*, MRA_*2374*, MRA_*2375* and *PPE71*. An IS*6110* element was located in the intergenic region between MRA_*2374* and *PPE38*, 21 bp upstream of *PPE38*. A 3 bp duplication of the target sequence (GCT) indicated direct integration. In addition, an indel event was identified 44 bp upstream of IS*6110* between the IS and MRA_*2374*. Twenty-eight bp had been deleted and replaced with a 33 bp sequence that corresponded to the 5’-untranslated region of *PPE71*, positions -63 to -95. This region of *PPE71* contains the PPE38F primer binding site and the PPE38F/R PCRs of this isolate thus produced amplicons smaller than expected for an IS*6110* insertion mutation. The 3 KZN whole genome sequences matched these findings apart from 1 SNP in KZN 4207 described in the main text. Two isolates from the same cluster as SAWC 2576 and 4 isolates from different F15 clusters were also analysed with the PPE38F/R PCR. Both isolates from the same cluster and 3 of the 4 isolates from different clusters produced an identical amplicon to SAWC 2576. The final isolate produced an amplicon representing the typical H37Ra-like genotype. We therefore conclude that the mutation described above is specific for most, but not all, F15 members.

**S7. SAWC 1815 (LAM, F11) and F11 (whole genome sequence)**

Sequencing revealed an intact copy of *PPE38* and a truncated copy of *PPE71*. The 3’ region of *PPE71* along with MRA_*2374* and MRA_*2375* had been replaced with an IS*6110* element. The resultant structure comprised the 5’ 258 bp of *PPE71*, followed by IS*6110* in reverse orientation, followed by *PPE38* including 81 bp of its 5’-untranslated sequence. IS*6110* did not produce a 3-4 bp duplication at the integration target sequence, indicating a recombination/deletion event. SAWC 1815 belongs to F11 (in PGG2), and *in silico* analysis of the whole genome sequence of another F11 isolate [71] confirmed these results. PCR analysis of 12 additional F11 isolates (3 from the same cluster as SAWC 1815 and 9 from different clusters) showed homology to SAWC 1815 in 11 cases. The remaining F11 isolate (from a different cluster) produced a PCR product slightly smaller than the 3 kb amplicon observed for all other F11 members. Sequence analysis of this amplicon revealed a 181 bp deletion due to IS*6110* being located at position 77 of *PPE71* instead of the usual position 258, as seen in all other F11 clusters. We were interested to know whether this mutation was cluster or isolate specific and therefore 7 additional isolates from this cluster were also analysed. Interestingly, none of the other isolates within this cluster had the same mutation; one isolate produced an amplicon representing the RvD7 genotype while the remaining 6 isolates failed to produce an amplicon. Further analysis of these isolates with the 21del PCR and a PCR using the plcA5’/PPE38F primer pair suggested that the isolates that failed to produce an amplicon retain *PPE38* but have undergone a (presumably IS*6110*-mediated) deletion that has removed the entire *PPE71* gene along with the upstream region that contains the PPE38F primer binding site. Our results therefore suggest the presence of at least 3 distinct *PPE38*/*71* genotypes within this single F11 cluster.

**S8. SAWC 3100 (PGG2, LAM, F14)**

This isolate failed to produce a PCR amplicon with the PPE38F/R, PPE38IntF/IntR and 21del (Fig 4) primer pairs. A PCR using primers predicted to amplify a product spanning from *plcA* to *PPE40* was also attempted but was unsuccessful. Other non-*PPE38*-related PCRs were successfully performed on this isolate and we therefore conclude that the negative results are due to complete deletion of the *PPE38* region. Two additional isolates from the same cluster as SAWC 3100 and 4 isolates from different F14 clusters were also analysed using the PPE38F/R PCR. Both isolates from the same cluster and 2 of the 4 isolates from different clusters also failed to produce an amplicon. The other 2 isolates produced an amplicon representing the RvD7 genotype (Fig 2a). We conclude that F14 can be divided into 2 lineages – those possessing the RvD7 genotype (representing the ancestral F14 clusters) and those lacking the entire *PPE38* region.

**S9. SAWC 1595 (PGG2, Quebec/S, F28)**

The PPE38F/R PCR produced an amplicon representing the RvD7 genotype (Fig 2a) which was confirmed by sequencing. A PCR using the PPE38IntF/IntR primers failed to produce a product, further confirming an RvD7 genotype. Eleven additional F28 isolates, including 3 from the same cluster as SAWC 1595, were analysed by both the PPE38F/R and PPE38IntF/IntR PCRs. Nine of these (including the 3 from the same cluster) produced identical results to SAWC 1595. The remaining 2 isolates revealed the ancestral H37Ra-like genotype suggesting that members of these 2 clusters diverged from other F28 clusters prior to the RvD7 mutation.

**S10. SAWC 1748 (PGG2, Pre-Haarlem, F24)**

The PPE38F/R PCR produced an amplicon representing the RvD7 genotype (Fig 2a) which was confirmed by sequencing. A PCR using the PPE38IntF/IntR primers failed to produce an amplicon further confirming an RvD7 genotype.

**S11. SAWC 1127 (Haarlem-like, F6)**

This isolate failed to produce an amplicon with the PPE38F/R PCR but produced a normal H37Ra-like amplicon with the PPE38IntF/IntR PCR. 21del analysis (Fig 4) in conjunction with these results suggested loss of the 3’ end of *PPE38*. We hypothesized that these results were best explained by the deletion of the 3’ end of *PPE38* (including the downstream region containing the PPE38R primer binding site) by an IS*6110*-associated recombination event. A PCR using the PPE38IntF/IS5’ primer pair produced an amplicon of approximately 1.7 kb conforming to our hypothesis. Sequence analysis of this amplicon showed that *PPE38* was truncated at position 417 by IS*6110*. PCR analysis of 3 additional isolates from the same cluster as SAWC 1127 as well as 3 isolates from different F6 clusters gave the same results, suggesting that this mutation is F6 specific.

**S12. SAWC 103 (PGG2, Haarlem-like, F7)**

PCR analysis of this isolate using the PPE38F/R, PPE38IntF/IntR and 21del primer pairs produced identical findings to sample SAWC 1127 described above. These findings were replicated in the analysis of 3 additional isolates from the same cluster as sample SAWC 103 as well as 5 isolates from different F7 clusters. Samples SAWC 103 and SAWC 1127 come from closely related lineages (F6 and F7, both designated as “Haarlem-like” – N. C. Gey van Pittius et al., manuscript in preparation) and we hypothesized that they would share the same IS*6110*-associated mutation (see S11). However, unlike the F6 isolates, none of the F7 isolates analysed produced an amplicon with the PPE38IntF/IS5’ PCR. PPE38IntF/Xho1 PCRs were also negative, indicating that the IS had not reversed direction (as seen when comparing Beijing lineages 27 and 29). We conclude that, as in F6 members, the 3’ region of *PPE38* is missing due to an IS*6110*-mediated recombination event, but that the IS*6110* element has subsequently been lost. It is possible that the F6 genotype is parental to F7 and that a further lineage-specific mutation involving the removal of the IS element has occurred.

**S13. SAWC 1645 (PGG2, Haarlem, F10)**

The PPE38F/R PCR produced an amplicon representing the RvD7 genotype. This was confirmed by sequencing the amplicon. However, a PCR using the PPE38IntF/IntR primers produced an amplicon of approximately 1100 bp. Despite several attempts, sequence analysis of this amplicon was unsuccessful due to multiple sequence signals. Additional analysis of 4 isolates from the same cluster as 1645 and 2 isolates from different F10 clusters produced the same amplicons indicating that the mutation is F10 specific. Sequencing of the 1100bp amplicon from one of these additional isolates was attempted but was also unsuccessful. The exact nature of the F10 *PPE38/71* gene structure therefore remained unresolved although further analysis of this sample with the 21del PCR suggests a single intact *PPE71* gene (Fig 4).

**S14. SAWC 1841 and Haarlem (whole genome sequence) (PGG2, Haarlem, F4)**

The PPE38F/R PCR produced an amplicon representing the RvD7 genotype (Fig 2a) which was confirmed by sequencing. A PCR using the PPE38IntF/IntR primers failed to produce a product further confirming an RvD7 geneotype. Analysis of the Haarlem whole genome sequence [71] indicated that the sequenced isolate corresponds to F4. This sequence also revealed an RvD7 genotype.

**S15. SAWC 2185 (Haarlem, F2)**

Sequence analysis revealed the typical H37Ra-like structure (Fig 2b) with the inclusion of IS*6110* at position + 51 of *PPE38* . A 4 bp duplication of the target sequence (GGAT) was observed. Note that the integration site is identical to that observed in the Beijing isolates (see above - lineages 31, 27 and 29) although in the case of the Beijing isolates an IS*6110*-associated recombination event with deletion, rather than direct integration, is responsible. Twelve other F2 isolates (4 from the same cluster as SAWC 2185 and 8 from different clusters) were analysed using the PPE38F/R PCR. All samples produced the same size amplicon as SAWC 2185 indicating that the mutation is F2-specific. This isolate was also found to contain the 21bp deletion (21del) in *PPE71* (see main text and isolates S29 and S30).

**S16. SAWC 2901 (PGG3, F16)**

The PPE38F/R PCR produced an amplicon representing the RvD7 genotype. This was confirmed by sequencing the amplicon. However, PCR using the PPE38IntF/IntR primers produced a 3.1 kb amplicon. Sequencing of this amplicon revealed an IS*6110* element 4 bp upstream from the start codon of MRA_*2375*. However, attempts to sequence the region between the IS and *PPE38* were unsuccessful. The reason for this is unclear but might involve deletion of the 3’ end of the IS. The exact nature of the *PPE38* region in this isolate and the reason for the conflicting PCR results thus remain unresolved although the PPE38IntF/IntR PCR suggests that both the *PPE38* and *PPE71* genes are present and intact.

**S17. SAWC 1608 (T-clade, F5)**

Sequence analysis revealed the typical H37Ra-like *PPE38/71* structure with the inclusion of IS*6110* at position + 84 of MRA_*2374*. A 3 bp duplication (GAG) at the site of integration indicates direct insertion. Only one other F5 member of a different cluster was analysed by PPE38F/R PCR due to the small size of the lineage. Results from this isolate revealed the RvD7 genotype and indicate that the 1608 mutation is not F5 specific.

**S18. SAWC 1956 (T-clade, F17)**

Sequence analysis revealed the typical H37Ra-like *PPE38/71* structure with the inclusion of IS*6110* at position + 330 of *PPE38*. The IS was orientated in the reverse direction to *PPE38*. A 4 bp duplication (GCGG) at the site of integration indicated direct insertion. Seven other F17 isolates (4 from the same cluster and 3 from different clusters) were analysed using the PPE38F/R PCR and all produced the same amplicon as SAWC 1956 indicating that this mutation is probably F17-specific.

**S19. T17 (PGG1, EAI) whole genome sequence**

Analysis of the PPE38 region revealed the RvD7 genotype (Fig 2a).

**S20. EAS054 (PGG1, EAI) whole genome sequence**

Analysis of the PPE38 region revealed the RvD7 genotype (Fig 2a).

**S21. T92 (PGG1, EAI) whole genome sequence**

In the T92 genome a BLAST search failed to identify any DNA sequences homologous to *PPE38/71*, MRA_*2374* or MRA_*2375*. Closer analysis revealed a 10.9 kb deletion (compared to the CDC1551 genome) spanning the region from *plcC* position 195 to *PPE40* position 1582 (Fig 3). Thus, along with the truncations of these 2 genes, the following genes are deleted: *plcB, plcA, PPE38,* MRA_*2374,* MRA_*2375, PPE71* and *PPE3*9. At the deletion point between *plcC* and *PPE40* a 158 bp sequence was observed that corresponded with *plcC* position 32 – 189 in reverse orientation. CDC1551 was used as a comparison here since, unlike H37Rv and H37Ra, it does not contain any IS*6110*-associated mutations within this region.

**S22. 94_M4241A (PGG1, atypical Beijing, Pre-F31, 27) whole genome sequence**

Although 94_M4241A belongs to the atypical Beijing lineage, it appears to have diverged from the atypical Beijing lineage before the evolution of F31 and F27 (the atypical Beijing lineages used in our clinical cohort). The reason for this assumption is that although this isolate contains the RD105 deletion, which is a marker for all Beijing lineage strains [83], it does not contain RD207 or RD181 (markers for F31 and F27, respectively – see figure S22 below). In isolate 94_M4241A, a BLAST search failed to identify any DNA sequences homologous to *PPE38/71*, MRA_*2374* or MRA_*2375*. Further analysis revealed a large deletion that removed 6.9 kb (compared to the CDC1551 sequence) spanning the region from *plcB* position 794 to 178 bp downstream from the 3’ end of *PPE39*, and its replacement with an IS*6110* element (Fig 3). In addition, alterations to the adjacent *PPE39* and *PPE40* genes were observed (Table 4). These are discussed in the main text.

**Figure S22.** **Position of Beijing lineage isolates in Beijing family tree:** Schematic representation of the positions of the Beijing lineage isolates used in this study in the Beijing family tree, based on presence and absence of deletions (RD’s) and IS insertions (IS) [56,82,83] .

**Atypical Beijing**

**Typical Beijing**

**RD105**

**RD207**

**RD181**

**RD142**

**RD150**

**IS21-5 / -19**

**94_M4241A**

**02_1987**

**F27 (SAWC 2701)**

**F29 (T85)**

**F31 (SAWC 2088)**

**F29 (SAWC 2076)**

**IS21-16**

**S23 (i). 02_1987 (atypical Beijing whole genome sequence)**

Similar to 94_M4241A above, strain 02_1987 also belongs to the atypical Beijing lineage, and it appears to have diverged from this lineage before the evolution of F31 and F27 (the atypical Beijing lineages used in our clinical cohort). The reason for this assumption is that although this isolate contains the RD105 and RD207 deletions, it does not contain the IS*6110* insertion IS21-16 or the RD181 deletion (putting it at a position pre-F31 and F27 – see figure S22, above). BLAST analysis of the *PPE38* sequence produced 3 hits. Closer inspection of the whole genome sequence revealed numerous and extensive alterations. From the left hand side of the figure below, normal sequence representing the Rv*1927* and Rv*1928c* genes is observed (white facing arrows). Gene Rv*1929c* (usually over 450 kb from the *PPE38* region) is truncated due to an IS*6110* insertion. Immediately 5’ of IS*6110* is sequence corresponding to the upstream region of *PPE71* and this gene and the 5’ region of MRA_*2375* then follow in the reverse direction to normal. MRA_*2375* is truncated at position 233 and the sequence following corresponds to the 3’ end of *MMPL9* (Rv*2339*, yellow arrow). Then follows over 15 kb of sequence (double headed arrow) containing around 10 genes normally found downstream of the *PPE38* region and including a large deletion that has fused *plcA* and *plcB* upstream of *PPE38* (not shown, see Table 3). *PPE38* has been disrupted by direct integration of IS*6110*. Following *PPE38* normal sequence resumes containing intact copies of MRA_*2374*, MRA_*2375* and *PPE71*. Following *PPE71* the vertical black line represents a recombination point where *PPE71* upstream sequence position -95 reverts to *PPE38* upstream sequence corresponding to position – 97. The *PPE38* upstream sequence continues to MRA_*2374* which is missing the first 58 bp due to a fusion with the 5’ end of *MMPL9* (yellow block).

**(ii) Simplified description of a major structural alteration in isolate 02_1987**

The most significant mutational event in terms of structural alteration of the 02_1987 genome involves the excision of an approximately 432 kb region spanning the genes Rv*1929c* to Rv*2339* (*MMPL9*) (grey shaded bar, top figure). This DNA segment has been reinserted, in reverse orientation, approximately 28 kb away. The IS6*110* element that disrupts Rv*1929c* (see S23 (i) above) appears to have recombined with an IS*6110* located at *PPE40* position + 47 resulting in a Rv*1929c* (truncated)/IS*6110*/*PPE40* (truncated) structure. The yellow bar in this Figure represents the genome region described in S23 (i), above, which contains numerous other macro-mutational events.

Normal genome sequence

Rv*1929c*

Rv*2339*

(*MMPL9*)

MRA_*2374*

Approx 432 kb region excised and reinserted in reverse direction

Rv*1929c*/IS*6110*/*PPE40*

Rv*1929c*/IS*6110*

MRA_*2374*/Rv*2339*

(*MMPL9*) fusion

See Fig 2m (i) above for detail

**S24. T85 (PGG1, typical Beijing F29).**

This isolate is a representative of the typical Beijing F29 lineage (see figure S22). Blast analysis of the genome showed a single *PPE38/71* copy and the absence of MRA_*2374* and MRA_*2375*. An IS*6110* element, in reverse orientation, was located at position 77 of *PPE71*. However, the sequencing appears to be unfinished since approximately 650 bp into the IS a long series of N’s is encountered before the sequence resumes revealing the 3’ end of *PPE38*. We were therefore unable to do a complete characterization of this region although available results confirm those obtained from our own typical Beijing F29 isolate SAWC 2076, (S4).

**S25. Strain C (LCC, “3 bander”, whole genome sequence)**

Analysis revealed the RvD7 genotype (Fig 2a). The single gene possesses an identical 21 bp deletion to *PPE71* in CDC1551 (red vertical line, see S26, below), demonstrating that *PPE71* has been retained and *PPE38* deleted in this case.

**S26. CDC1551 (LCC, “4 bander”, whole genome sequence)**

The CDC1551 *PPE38* region is identical to that of H37Ra apart from the presence of a 21 bp deletion (21del) in *PPE71* (red vertical line). This in-frame deletion is predicted to result in the loss of amino acids 357 - 63 (GGAGAGM). CDC1551 is a member of the PGG2 low IS*6110* copy clade (LCC) and possesses 4 IS*6110* elements in its genome (“4 bander”). The 21del mutation is also found in the *M. tuberculosis* strain C (another LCC member, “3-bander”) whole genome sequence (see S25) and its presence was also investigated in other LCC and PGG2 members of our cohort (see main text and Figs 4 and 5).

**S27. *M. bovis* and *M. caprae* whole genome sequence**

In *M. bovis* and *M. caprae*, *PPE38/71* is within the RD5 region which spans the genes *esxO* position 104 to *PPE39* position 1358 [23-25] (Fig 3). The whole genome sequences of these organisms [75] thus lacks any region homologous to *PPE38/71*.

**S28. *M. bovis* (BCG) Pasteur 1173P2 (whole genome sequence)**

*M. bovis* (BCG) Pasteur 1173P2 has an identical RD5 region to *M. bovis* and thus also lacks any region homologous to *PPE38*. Unlike *M. bovis* it also contains an additional deletion that affects the *PPE39* and *PPE40* genes. This is described in the main text and Table 4.

**S29. *M. microti* OV254 (whole genome sequence)**

*M. microti* diverged from the *M. bovis* lineage before the *M. bovis*-specific RD5 deletion [23]. However, *M. microti* OV254 has undergone a similar deletion named RD5^mic^ that spans a 9 kb region (compared to CDC1551) from *plcC* position 867 to *PPE39* position 325 [32] (Fig 3). RD5^mic^ was missing from the vole isolates of *M. microti* only and was present in *M. microti* strains isolated from humans [32]. The sequenced *M. microti* strain [77] was isolated from voles and a BLAST analysis of its genome thus failed to locate an *M. tuberculosis PPE38* homologue.

**S30. Oryx bacillus**

The oryx bacillus diverged from the *M. bovis* lineage before the *M. bovis*-specific RD5 deletion [23]. However, the oryx bacillus has undergone a similar, but specific, deletion of the *PPE38/71* region named RD5^oryx^, which has caused the deletion of 8.3 kb kb (compared to CDC1551), spanning *plcB* position 260 to *PPE40* position -6 (Fig 3). An IS*6110* element is located at the point of deletion. [23].

**S31. Dassie bacillus**

The dassie bacillus diverged from the *M. bovis* lineage before the *M. bovis*-specific RD5 deletion [23]. However, dassie bacillus has undergone another similar, but specific, deletion of the *PPE38* region annotated as RD5^das^ [22] (Fig 3). RD5^das^ deletes approximately 9.8 kb, spanning from 105 bp downstream of *plcC* to an unidentified position downstream of *PPE40*. An IS*6110* insertion sequence in its place.

**S32. *M. africanum* isolates K85, CPHL_A and GM041182 (whole genome sequences)**

Based on genomic deletion analysis, *M. africanum* can be subdivided into 2 distinct subtypes, subtype 1(a) and 1(b), both of which diverged from the *M. bovis* lineage before the *M. bovis*-specific RD5 deletion [23]. Three *M. africanum* strains have been sequenced, namely K85, CPHL_A and GM041182. CPHL_A belongs to the *M. africanum* subtype 1(b) sublineage 2 lineage due to the RD713 and RD711 deletions in its genome, while K85 and GM041182 belong to the *M. africanum* subtype 1(a) sublineage 2 and 3, respectively, due to the RD701 deletion and polymorphisms in their *rpoB* genes. Analysis of the *PPE38* region of their respective genomes showed that CPHL_A possessed the RvD7 genotype (Fig 2a) while the other isolates showed the ancestral H37Ra-like genotype (Fig 2b). Isolate K85 also possessed a 6 bp deletion in *PPE38* that is predicted to result in incorrect amino acid incorporation from position 352 and premature termination.
